# Ultrafast ROS Scavenging Activity of Amur Maple Tree Extracts Confers Robust Cardioprotection for Myocardial Ischemia/Reperfusion Injury

**DOI:** 10.3390/antiox14060671

**Published:** 2025-05-31

**Authors:** Aoyang Pu, Woo-Sup Sim, Yuen-Kei Liem, Yimin Lai, Bong-Woo Park, Kyoung-Tae Lee, Hun-Jun Park, Kiwon Ban

**Affiliations:** 1 Department of Biomedical Sciences, City University of Hong Kong, Kowloon, Hong Kong SAR, China; aoyangpu2-c@my.cityu.edu.hk (A.P.); ykliem2-c@my.cityu.edu.hk (Y.-K.L.); yiminlai2-c@my.cityu.edu.hk (Y.L.); 2 Tung Biomedical Sciences Centre, City University of Hong Kong, Hong Kong SAR, China; 3 Department of Biomedicine & Health Sciences, The Catholic University of Korea, Seoul 06591, Republic of Korea; woosup269@naver.com; 4 Department of Biomedical Informatics, College of Applied Life Sciences, Jeju National University, Jeju 63243, Republic of Korea; bwpark@jejunu.ac.kr; 5 Forest Microbiology and Application Division, National Institute of Forest Science, Suwon 16631, Republic of Korea; leekt99@korea.kr; 6 Division of Cardiology, Department of Internal Medicine, Uijeongbu St. Mary’s Hospital, Uijeongbu 11765, Republic of Korea; 7 Cell Death Disease Research Center, The Catholic University of Korea, Seoul 06591, Republic of Korea

**Keywords:** ischemia-reperfusion injury, anti-ROS, anti-ferroptosis, cardioprotection

## Abstract

*Ginnalin A* (GA), a polyphenolic compound derived from amur maple trees, has been identified as a powerful scavenger of reactive oxygen species (ROS). Recognizing the pivotal role of ROS in exacerbating secondary damage during myocardial ischemia-reperfusion injury (MIRI), we fractionated GA-enriched extracts from the leaves of the amur maple tree, *Acer tataricum* L. subsp. *ginnala* (*Maxim.*) *Wesm.*, using common solvents of dichloromethane (DCM) and ethyl acetate (EA). When co-administered for 30 min, the DCM- and EA-fractioned extracts effectively protected cardiomyocytes from H_2_O_2_-induced damage. ROS-sensitive probes indicated that treatment with *ginnala* extracts significantly reduced both intracellular and mitochondrial ROS levels. Instead of enhancing the activity of antioxidative enzymes, the *ginnala* extracts acted as natural antioxidases, directly scavenging various ROS such as superoxide, H_2_O_2_, hydroxyl radical, and Fe^2+^ within just 20 min. In a MIRI rat model, the in vivo administration of *ginnala* extracts provided significant cardioprotection by preserving viable myocardia and enhancing cardiac functions. Additionally, treatment with *ginnala* extracts significantly reduced cardiac fibrosis and denatured collagen. Our study suggests that the ultrafast ROS scavenging capability of *ginnala* extracts offers substantial heart protection during MIRI. Incorporating *ginnala* extracts as a pharmacological intervention during reperfusion could effectively mitigate ROS-induced cardiac injury.

## 1. Introduction

Coronary artery disease (CAD) remains a pressing global health issue that is responsible for a substantial number of worldwide fatalities [1]. Myocardial infarction (MI), a primary complication of CAD, contributes significantly to morbidity and mortality rates, and there is a rising trend in related deaths. MI arises when the coronary artery, supplying blood to the heart muscle, becomes obstructed, resulting in cardiomyocyte death and impaired cardiac function. Timely treatment, ideally within the first hour of symptom onset, is critical for improving patient outcomes, while reperfusion therapy, aimed at restoring blood flow to the affected heart region, is a crucial emergency intervention that is achieved through thrombolysis or primary percutaneous coronary intervention (PCI) [2]. However, reperfusion can paradoxically lead to myocardial ischemia/reperfusion injury (MIRI) and thus exacerbate tissue damage.

Various strategies, such as ischemic preconditioning, where the heart is exposed to short periods of ischemia before an MI occurs, have been extensively investigated for the prevention of MIRI [3]. Although promising results were obtained from preclinical studies using animal models, these strategies have limited clinical value due to the unpredictable nature of ischemia and potential secondary damage from invasive procedures. Pharmacological interventions during reperfusion have also shown potential for cardioprotection, yet the clinical utility of these approaches is limited since the availability of drugs for co-administration in MI patients is restricted [4]. Hence, exploring alternative therapeutic avenues is of great significance to enhancing outcomes in clinical patients with MIRI.

During reperfusion, the restoration of blood flow leads to mitochondrial damage and electrolyte imbalances, resulting in the rapid production of reactive oxygen species (ROS), such as hydrogen peroxide (H_2_O_2_) and hydroxyl radical (•OH), which contain unpaired electrons [5]. The excessive accumulation of ROS can cause damage to cell and organelle membranes, which leads to programmed cell death through apoptosis or autophagy [6]. ROS can also induce lipid peroxidation, which disrupts the membrane structure and releases toxic substances. Given the adverse role of ROS in MIRI, researchers have intensively explored the application of antioxidants as potential therapeutic agents. However, despite evidence that indicates cardioprotection from many natural and synthetic antioxidants, the clinical use of antioxidase-like drugs such as trimetazidine and superoxide dismutase (SOD) has not significantly improved heart function in patients [7]. This underscores the importance of identifying potent antioxidants that are capable of efficiently eliminating ROS to provide timely cardioprotection during MIRI.

Recent studies have focused on phenolic compounds, as they are promising antioxidants due to their ability to chelate ions and scavenge free radicals [8]. Extracts of leaves from *ginnala*, a species of amur maple tree, in particular, have been reported to have exceptional antioxidative activities, offering biological benefits in attenuating inflammation and tissue damage caused by oxidative stress [9,10]. *Ginnalin A*, a major component of *ginnala* leaf extracts, exhibited the rapid inhibition of lipid peroxidation, the scavenging of hydrogen peroxide, and the elimination of free radicals in a few organs but has never been studied in the cardiovascular system [11]. *Ginnalin A* contains two galloyl derivatives, which allows it to efficiently and rapidly sense and diminish environmental ROS. These findings highlight the remarkable antioxidative properties of *ginnala* extracts, particularly those that are due to the action of *Ginnalin A*. The rapid and efficient elimination of ROS could offer promising prospects for readily available and valid therapeutics for combating MIRI and other conditions that involve excessive oxidative stress.

Thus, in the present study, we aimed to investigate the cardioprotective effects of *ginnala* leaf extracts (hereafter referred to as *ginnala* extracts) against MIRI by harnessing their remarkable antioxidative properties. Our results demonstrated that responsive components within *ginnala* extracts rapidly and effectively neutralize ROS surges within seconds in a sequential manner, safeguarding cardiomyocytes from MIRI. Through our investigation, which involved ROS measurement, we observed a significant and rapid decrease in intracellular, mitochondrial, and extracellular ROS levels upon concurrent treatment with *ginnala* extracts and various ROS inducers. Moreover, experiments conducted in a rat model of MIRI showcased notable enhancements in cardiac function, reduced infarct sizes, and the mitigation of fibrosis and cardiac remodeling following the administration of *ginnala* extracts. These results highlight the antioxidative and cardioprotective capabilities of *ginnala* extracts, particularly emphasizing *Ginnalin A*’s proficiency in countering ROS.

## 2. Materials and Methods

### 2.1. Leaf Materials

*Acer tataricum* L. subsp. *ginnala* (*Maxim.*) *Wesm.* were cultivated in the reservatory of forest biomaterials of the Korea Forest Research Institute, Jinju, Korea (TM coordinates; X: 305,331, Y: 191,057) and identified at Forest Biomaterials Research Center (Jinju, Korea). Voucher specimens were preserved by the Korea Forest Service (NF-A2-ATg-L). The leaves were collected and shade-dried for 10 days.

### 2.2. Extraction, Isolation, and of Acertannin

Extraction of the dried leaves (1 kg) was conducted three times, for two days each time, using EtOH (10 L) at room temperature (RT), followed by evaporation under reduced pressure to yield approximately 400 g extracts. A part of the extract (~167.0 g) was then processed on Sephadex LH-20 (10–25 μm, 10 cm × 80 cm, GE Healthcare Bio-Science AB, Uppsala, Sweden) and eluted successively with equal volume of chloroform and methanol to produce 9 fractions (1~9, Figure S1), where fraction 2 (2.6 g, 40% eluate) was put through ODS-A (30–50% MeOH) and ODS-A (25–35% ACN). Fraction 2-6-11 (2.4 g, 60% eluate) was determined to be acertannin (740 mg). The solvents, such as DCM and EA, were fully evaporated, and DMSO was used to dissolve the dried extracts prior to cell treatment and in vivo administration to rat hearts.

### 2.3. Neonatal Rat Cardiomyocytes (NRCMs) Isolation

NRCMs were isolated from neonatal Sprague-Dawley (SD) rat (2–4 days) hearts in accordance with institutional guidelines. The procedure was approved by the Institutional Animal Care and Use Committee of The Catholic University of Korea (approval no. CUMC-2024-0279-03; approval date: 1 December 2024). Generally, rat heart ventricles were collected, chopped, and digested using 0.1% trypsin solution (Gibco, 25200056, Grand Island, CA, USA) with gentle agitation at 4 °C in a fridge for 16 h. The tissues were further incubated with 1 mg/mL rat type 2 collagenase (Worthington, LS004174, Lakewood, NJ, USA) at 37 °C for 20 min, and this step was repeated 3 times in total. After digestion, the supernatant was collected and transferred to cold DPBS (Gibco, 14190144, USA). A Percoll (Cytiva, Marlborough, MA, USA) density-gradient centrifugation was performed for 30 min at 1800× *g* at 4 °C to remove non-cardiomyocytes, and obtained NRCMs were seeded in gelatin (Sigma, G1393, Darmstadt, Germany)-coated cell culture dishes with a seeding density of 1 × 10^5^ per cm^2^.

### 2.4. Cell Culture

Rat cardiac myoblast H9c2 cell line (ATCC, Manassas, VA, CRL-1446, USA) and NRCMs were used in all experiments. H9c2 cells were cultured with high-glucose Dulbecco’s Modified Eagle’s Medium (DMEM, Gibco, 11965092, USA) supplemented with 10% fetal bovine serum (FBS, ExCell, FSP500, Shanghai, China) (Gibco, 26140079, USA), 1% non-essential amino acid (Gibco, 11140050, USA), 1% antibiotics (Gibco, 15240062, USA), and 1% sodium pyruvate (Gibco, 11360070, USA). Prior to *ginnala* leaf extract treatment, H9c2 cells were plated on 96-well plates with a seeding density of 1 × 10^5^ per cm^2^ and cultured overnight to allow cell attachment. NRCMs were maintained with low-glucose DMEM (Gibco, 10567014, USA), together with 5% horse serum (HS, Gibco, 16050122, USA), 1% non-essential amino acid, and 1% antibiotics. Both H9c2 cells and NRCMs were cultured 37 °C under 5% CO_2_.

### 2.5. CCK-8 Assay

For the H_2_O_2_-induced cell injury, H9c2 cells and NRCMs were either co-treated with H_2_O_2_ (Sigma-Aldrich, H1009, Darmstadt, Germany) and *ginnala* leaf extract for 30 min or pre-treated with *ginnala* leaf extract candidates for 1 h prior to 30 min of exposure to H_2_O_2_. To induce ferroptosis, ferroptosis inducers, either Erastin (S7242, Selleck, Boston, MA, USA) or RSL3 (Sigma-Aldrich, SML2234, Germany), were co-administrated with *ginnala* leaf extract on the cells for 12 or 24 h. After administration, CCK-8 assay (Dojindo, CK04, Tokyo, Japan) was performed to evaluate the cell survival. In brief, the CCK-8 reagent was mixed with FBS-free culture medium and incubated with the cells at 37 °C in an incubator for 2 h. Subsequently, the absorbance was measured at 450 nm.

### 2.6. Lactate Dehydrogenase (LDH) Assay

Lactase dehydrogenase (LDH) assay (Cell Biolabs, CAB241, San Diego, CA, USA) was used to detect the cellular dagame by using culture medium. Briefly, after treatment, as mentioned previously, 90 µL of supernatant from various groups was mixed with 10 µL of LDH reagent. After 1 h incubation at 37 °C, the absorbance was measured at 450 nm. To obtain accurate measurements, the absorbance of the media-only controls was subtracted from the results of each group.

### 2.7. Live and Dead Assay

The live and dead assay (live/dead assay) was conducted according to the manufacturer’s instructions (Invitrogen, L3224, Waltham, MA, USA). Briefly, after treatment, calcein AM and EthD-1 were mixed with phenol-free DMEM (Sigma-Aldrich, D5921, Burlington, MA, USA) to final concentrations of 2 μM and 4 μM, respectively. Thirty minutes after incubation at RT, the stained cells were imaged and analyzed using a fluorescence microscope (Nikon, Tokyo, Japan). The number of live and dead cells from each group were quantified and normalized using the values of controls by ImageJ (NIH, Bethesda, MD, USA).

### 2.8. Measurement of Cellular and Mitochondrial ROS Level

Prior to treatment, 5 µM DCFDA (Sigma-Aldrich, D6883, USA) solution was added to stain the H9c2 cells and NRCMs. After 45 min incubation at 37 °C in the dark, the solution was then removed and replaced with PBS. After treatment with H_2_O_2_ and *ginnala* leaf extracts, the cellular DCF fluorescence intensity of cells was measured immediately at 488/530 nm. The cellular ROS level was also examined using flow cytometry, for which treated cells were collected using 0.1% trypsin to conduct flow cytometer analysis. Mitochondrial ROS was measured and analyzed by a kit that indicated mitochondrial superoxide (the MitoSOX^TM^ Red, Invitrogen, M36008, USA). In brief, cells were stained with 5 μM MitoSOX^TM^ reagent for 10 min at 37 °C followed by rinsing with PBS twice. Subsequently, cells were treated with H_2_O_2_ and *ginnala* leaf extracts for 30 min before the fluorescent intensity was determined at 529/599 nm.

### 2.9. Enzymatic Assays

After treatment, NRCMs were harvested by cell scalper and lysed using lysis buffer (Beyotime, P0013, Nantong, China) with 1x Protease Inhibitor Cocktail (MCE, HY-K0010, New York, NY, USA). The protein concentration of cell lysates from each treatment group was determined by Pierce^TM^ BCA Protein Assay Kit (Thermo Scientific, 23227, Logan, UT, USA). The malondialdehyde (MDA, Beyotime, S0131, China) contents, enzymatic activities of glutathione peroxidase (GPx, Beyotime, S0056, China), superoxide dismutase contents (SOD, Beyotime, S0101, China), catalase (CAT, Beyotime, S0051, China), and total, reduced, and oxidized glutathione (GSH/GSSG, Beyotime, S0053, China) were detected following instructions from the reagents manufacturers.

### 2.10. DPPH Assay

The protocol for determining the scavenging activities of extracts by using 2,2-diphenyl-1-picrylhydrazyl (DPPH) assay was modified as previously reported. Briefly, DPPH (Sigma-Aldrich, D9132, USA) working compound was freshly prepared after 2 mg DPPH was dissolved in 54 mL methanol. DCM, EA extracts, L-ascorbic acid (Sigma-Aldrich, A4544, Germany), and NAC (Sigma-Aldrich, A7250, Germany) were mixed with DPPH solution. Beginning immediately after the reaction, the absorbance was measured at 515 nm every 3 min until 30 min.

### 2.11. Hydrogen Peroxide (H_2_O_2_) Assay

Hydrogen peroxide was quantified using hydrogen peroxide assay (Elabscience, E-BC-F001, Wuhan, China). In brief, a standard curve was produced using H_2_O_2_ solution in quantities in the range of 0.5–10 µM. DCM and EA at selected concentrations were mixed with 700 µM H_2_O_2_ solution, followed by 30 min reaction, after which the fluorescence intensity of each group was determined at 535/587 nm.

### 2.12. Total Antioxidant Capacity Assay

Total antioxidative activity was measured with two total antioxidant capacity assay kits and using the ferric reducing ability of plasma (FRAP) (Beyotime, S0116, China) and [2,2′-azinobis (3-ethyl-benzothiazoline-6-sulphonate)] (ABTS) (Beyotime, S0119, China) methods, following the manufacturer’s instructions. For FRAP assay, quantities of FeSO_4_ solution ranging from 0.15 to 1.5 mM were included to yield the standard curve. DCM and EA at selected concentrations were mixed with 180 μL of FRAP working solution. Immediately after reaction, the absorbance was measured at 593 nm every 3 min for 35 min. FRAP activities of each sample were determined according to the equivalent value of FeSO_4_ concentration calculated and obtained based on the standard curve. For ABTS assay, quantities of Trolox ranging from 0.15 to 1.5 mM were employed to generate the standard curve. DCM and EA at selected concentrations were mixed with 200 μL of ABTS working solution. Immediately after reaction, the absorbance of each group was acquired at 734 nm every 3 min for 30 min. ABTS activities were assessed based on the equivalent value of Trolox concentrations.

### 2.13. Seahorse Metabolic Analysis

XF glycolysis stress, cell energy phenotype, and cell mitochondrial stress tests were determined to demonstrate the metabolic profile of *ginnala* extract-treated NRCMs. It should be noted that, unlike the 24 h incubation time used for drugs, to closely mimic the cell state from previous cell viability assays, the 30 min co-treatment of *ginnala* extracts with or without H_2_O_2_ solution was used as the protocol for cell treatment. One day prior to treatment, Agilent Seahorse XFe Analyzer (Agilent Technologies, Santa Clara, CA, USA) was turned on to warm it up, the cartridge was hydrated with XF Calibrant (1 mL/well), and placed in a 37 °C incubator without supply of CO_2_. Wave program was used to design the experimental flow. Different assay media were formulated for different tests. For glycolysis stress, assay medium was prepared using Base Medium supplemented with 1 mM glutamine. The medium containing 1 mM pyruvate, 2 mM glutamine, and 10 mM glucose was used for profiling cell energy phenotype and cell mitochondrial stress tests. A total of 0.1 N NaOH was required for pH adjustment of assay media to 7.4, after which assay media would be incubated in a 37 °C water bath prior to use. After treatment and before the assays were run, NRCMs from XF 24-well cell culture microplate were refreshed with warmed assay medium and placed into a 37 °C incubator without supply of CO_2_ for 1 h. Specific compounds were prepared and loaded into the appropriate ports of the hydrated sensor cartridges as follows: for glycolysis stress test: 56 µL glucose (10 mM), 62 µL oligomycin (1 µM), and 69 µL 2-DG were injected into ports A, B, and C, respectively; for cell energy test: 20 µL stressor mix containing 50 µM oligomycin and FCCP were injected into port A; for mitochondrial stress test: 56 µL oligomycin (15 µM), 62 µL FCCP (5 µM), and 69 µL Rot/AA (5 µM) were injected into ports A, B, and C, respectively. After loading compounds, sensor cartridges were be plated on the instrument for 15 min calibration, after which the designated program for each assay would start.

### 2.14. Structural Identification of Acertannin

Purity of acertannin was determined by a LC-q-ToF mass spectroscopy (Impact II, Bruker, Billerica, MA, USA) and ultra-performance liquid chromatography (Waters H-class system, Milford, MA, USA), which determined it to have approximately 98% purity. Spectral analyses were conducted to identify the structure of acertannin. Avance DRX500 (Bruker, USA) was employed to acquire the 1-D nuclear magnetic resonance (NMR) findings, such as 1H (500 MHz) and 13C (125 MHz) NMR, in Center for Research Facilities of Gyeongsang National University, Jinju, Korea. A Waters H-class UHPLC system interfaced with a Bruker Impact II hybrid quadrupole time of flight mass spectrometer (Bruker, USA) was used to analyze the samples. A Waters Acquity UPLC^®^ BEH C18 (150 × 2.1 mm 1.7 µm, Waters Corporation, Milford, MA, USA) was included to perform the chromatographic separations. This step was carried out at 35 °C with gradient elutions containing 0.1% formic acid in water (A) and 0.1% formic acid in methanol (B) as mobile phase with a flow rate of 0.2 mL/min. The elutions used included 20%, 0–3 min; 20–100%, 3–50 min; 100%, 50–535 min. The volume for injection of each sample was 5 μL. Mass spectrometry was conducted in negative electrospray ionization mode and the spectra were obtained via scanning the mass range from *m*/*z* 50 to 1500 using MS and MS/MS modes. Nitrogen was applied as a gas for drying, nebulizing, and collision and the drying gas flow rate was set as 8.0 L/min. Heated capillary temperature was at 200 °C while 0.8 bar was set as the nebulizer pressure. The source parameters, the capillary voltage (VCap) and source plate offset, were set at 3000 V and 500 V, respectively.

### 2.15. Experimental Animals

All procedures involving animals adhered to institutional and international ethical standards, including NIH regulations and the European Parliament’s 2010/63/EU directive. Prior approval for the study was granted by the IACUC of The Catholic University of Korea (Approval No. CUMC-2023-0234-01; Approval date: 1 October 2023).

### 2.16. Ischemic/Reperfusion Injury Model and In Vivo Drug Administration

To establish ischemia/reperfusion (I/R) injury, male Fischer 344 rats (age: 8 weeks; weight: 160–180 g) sourced from Central Lab. Animal Inc. were anesthetized with 2% isoflurane and ventilated following intubation via an 18-gauge catheter. The procedure was carried out on a preheated platform (37 °C). After thoracotomy and LAD exposure, the artery was ligated with a 7-0 suture for one hour. Five minutes prior to reperfusion, extracts (EA: 168 µg/kg; DCM: 84 µg/kg in 50 µL volume) were injected at two peri-infarct zones. Reperfusion was achieved by releasing the ligature, and the chest was closed aseptically.

### 2.17. Measurement of Myocardial Infarct Size

Twenty-four hours post-reperfusion, myocardial damage was evaluated using Evans blue and TTC dyes. Following LAD re-occlusion and intravenous administration of 8% Evans blue, the heart was excised and sliced into transverse sections. The tissue was stained with 2% TTC at 37 °C for 10 min, then fixed with 4% paraformaldehyde. Infarct demarcation was visualized as blue (non-perfused), red (viable), and white (necrotic). ImageJ software (version 1.54g) was utilized to quantify infarct extent and the area at risk.

### 2.18. Evaluation of Heart Function by Echocardiography

The rats were lightly anesthetized with 2% isoflurane (Hana Pharm, Seongnam, Republic of Korea). Echocardiography was performed using a 15 MHz probe (Philips Affiniti 50G, Eindhoven, Dutch) under mild anesthesia. Recordings were obtained at 4 h, 1 week, and 2 weeks after surgery. Investigators were blinded to group allocation. Ejection fraction and fractional shortening were computed using the following equation:

EF (%) = [(LVEDV−LVESV)/LVEDV] × 100

FS (%) = [(LVEDD−LVESD)/LVEDD] × 100

### 2.19. Hemodynamic Measurements

At 2 weeks post-injury, invasive cardiac function analysis was conducted under anesthesia. After thoracotomy, a conductance catheter (2F, Millar SPR-838, Pearland, TX, USA) was inserted into the left ventricle. Pressure-volume loops were monitored using a system from Emka Technologies and converted via a PowerLab interface (ADInstruments, Colorado Springs, CO, USA). Load-independent indices such as ESPVR and EDPVR were derived by transient IVC occlusion. Parallel conductance calibration involved injection of 20% NaCl (60 µL) through the jugular vein, and LV blood samples were collected for volume determination.

### 2.20. Measurement of Capillary Density by Immunohistochemical Staining

Following overnight fixation in 4% paraformaldehyde, hearts were embedded in paraffin and sectioned at 5 µm thicknesses (Leica RM2255, Wetzlar, Germany). After dewaxing and hydration, heat-induced epitope retrieval was performed. Slides were incubated overnight at 4 °C with CHP (20 μM), anti-cTnT (1:200, Abcam, Cambridge, UK), and anti-CD31 (1:200, R&D). Secondary antibodies included Alexa Fluor 488 donkey anti-mouse IgG and Alexa Fluor 594 rabbit anti-goat IgG (1:400, Invitrogen, Waltham, MA, USA). DAPI-containing mounting medium was applied for nuclear staining. Cardiomyocyte and capillary counts, as well as collagen denaturation areas, were measured in five randomly selected regions per sample.

### 2.21. Measurement of Myocardial Infarct Size by Masson’s Trichrome Staining

Masson’s trichrome stain (Sigma HT15) was employed to assess fibrotic remodeling. Sections were fixed in Bouin’s solution at 56 °C, then sequentially stained using Weigert’s iron hematoxylin for 10 min, Biebrich scarlet acid fuchsin for 20 min, and aniline blue for 15 min. Blue and red staining indicated fibrotic and viable myocardium, respectively. Scanned images (Pannoramic MIDI) were analyzed using ImageJ to quantify fibrotic area.

### 2.22. Data Analysis

Statistical analyses were performed using GraphPad Prism 9 (GraphPad Software, La Jolla, CA, USA). Quantitative data are presented as mean ± standard error of the mean (SEM), unless otherwise specified. Depending on the data distribution and comparison groups, statistical significance was assessed using two-tailed Student’s *t*-tests or one-way ANOVA followed by Tukey’s post hoc test. Detailed information regarding the statistical methods used is provided in the respective figure legends.

## 3. Results

### 3.1. Preparation of ginnala Extracts Can Alleviate Cardiomyocyte Death from Oxidative Injury

**Figure 1:**
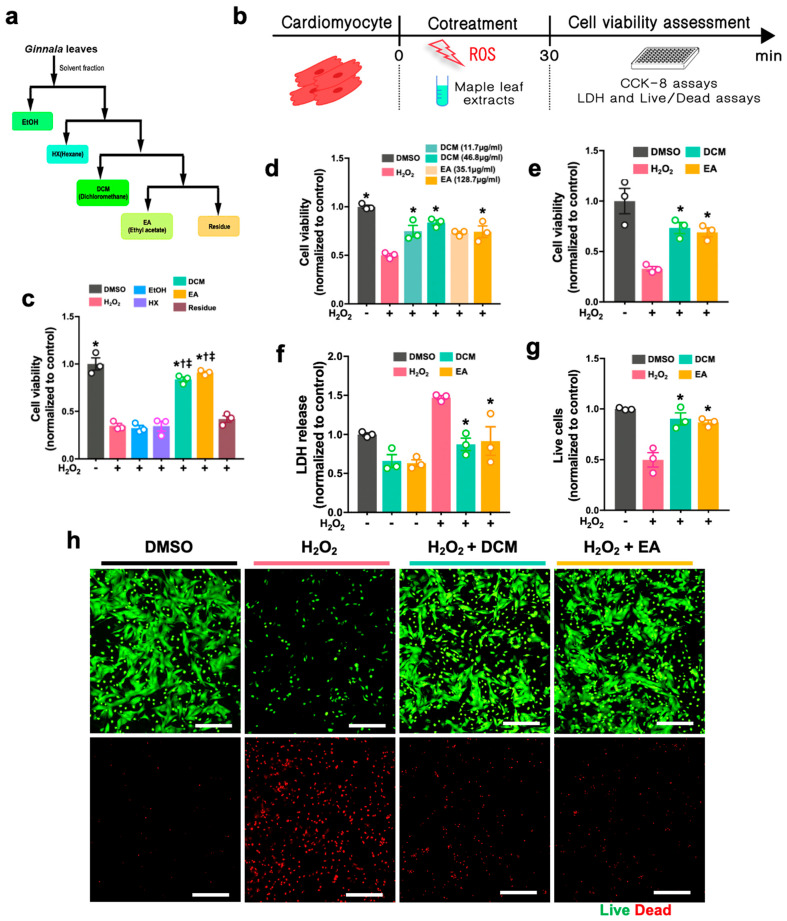
*ginnala* leaf extracts protected NRCMs against H_2_O_2_-induced cellular injury. (**a**) Experimental protocol for in vitro myocardial ischemia/reperfusion injury: co-treatment indicates 30 min incubation of extracts along with 600 μM H_2_O_2_ treatment. (**b**) Flowchart demonstrates a sequential fractionation and purification of *ginnala* leaf extracts. (**c**) Selection of *ginnala* leaf extracts candidates that showed the best cytoprotective effects in 30 min co-treatment with 600 μM H_2_O_2_, as determined by CCK-8 assay on H9c2 cells. Two *ginnala* leaf extracts (DCM and EA) exhibited higher cardioprotective effects than other fractions. * *p* < 0.05 versus H_2_O_2_; † *p* < 0.05 versus EtOH; ‡ *p* < 0.05 versus HX. *n* = 3. (**d**) Cytoprotective effects of extracts from two fractions (DCM and EA) in 30 min co-treatment with 600 μM H_2_O_2_ on H9c2 cells, as determined by CCK-8 assay. * *p* < 0.05 versus H_2_O_2_. *n* = 3. (**e**) DCM and EA improved NRCMs viability in 30 min co-treatment with 800 μM H_2_O_2_. (**f**) NRCM damage was evaluated by extracellular release of LDH and (**g**,**h**) live/dead assay. * *p* < 0.05 versus H_2_O_2_. *n* = 3. Scale bar = 200 μm. Data obtained from three independent experiments (with *n* = 3 for each experiment) are represented by the mean ± SEM.

Our primary goal was to evaluate the therapeutic potential of ginnala extracts against myocardial ischemia-reperfusion injury (MIRI). To achieve this aim, we first obtained fractions of extracts by harvesting and pulverizing fresh leaves from *Acer tataricum* L. subsp. *ginnala* (*Maxim.*) *Wesm.*, using 100% ethanol (EtOH) as the extracting solvent. The ethanol extract was then fractionated and purified using different organic solvents with varying polarities, including hexane (HX), dichloromethane (DCM), and ethyl acetate (EA). Additionally, a residue was collected as one of the treatment groups. In total, we obtained five fractions from the *ginnala* leaves to investigate their potential cardioprotective effects against MIRI (Figure 1a).

In an in vitro model in which oxidative stress was induced with H_2_O_2_ to simulate MIRI, we assessed the cytoprotective effects of these extracts. Importantly, each extract was tested using a co-treatment model where they were administered simultaneously with H_2_O_2_ for 30 min to mimic the speedy progressive conditions observed during MIRI in clinical settings (Figure 1b).

Initially, we used rat H9c2 cells, an immortalized rat cardiomyoblast cell line, for screening purposes. The CCK-8 cell viability assay revealed that H_2_O_2_ treatment led to rapid cell death, reducing the cell viability to less than ~50%. Interestingly, the DCM and EA fractions showed significant cytoprotective effects by increasing the cell viability to 83.7 ± 4.5% and 90.9 ± 2.5%, respectively (Figure 1c). Of note, given that the DCM and EA were completely evaporated, and only DMSO was employed to dissolve the extracts, the observed protection was unlikely to have been disturbed by the relevant solvents, but was majorly attributed to the active components from the *ginnala* extracts. Additional assays identified the most effective concentrations of the DCM and EA fractions in promoting cytoprotective effects against simulated MIRI (Figure 1d). Subsequent analyses such as CCK-8 (Figure 1e), lactate dehydrogenase (LDH) (Figure 1f), and LIVE/DEAD assays (Figure 1g,h) using neonatal rat cardiomyocytes (NRCMs) further verified the substantial cardioprotective effects of the screened DCM and EA fractions against H_2_O_2_-induced cell death. Additionally, these fractions were effective in improving the cell viability on both H9c2 cells and cardiomyocytes when a pre-treatment protocol was applied in which extracts were pre-administrated for 1 h prior to 30 min exposure to H_2_O_2_ (Figure S2), which indicated their prompt cellular uptake and intracellular protection capabilities. In summary, both the DCM and EA fractions exhibited ultrafast cytoprotective effects in the oxidative stress model.

### 3.2. ginnala Extracts Inhibited the Generation of Intracellular ROS

**Figure 2:**
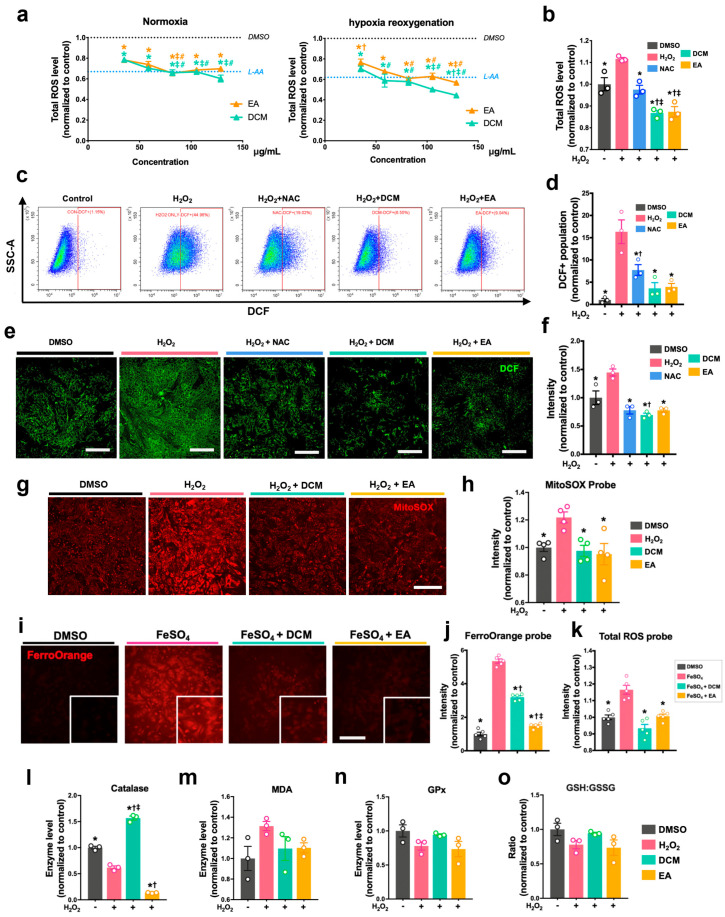
*ginnala* leaf extracts regulated intracellular oxidative stress. (**a**) Two fractions of *ginnala* leaf extracts, DCM and EA extracts with a series of concentrations, inhibited cellular ROS production in H9c2 cells under normoxia and hypoxia conditions after 30 min incubation. ROS levels were determined by DCFDA assay. * *p* < 0.05 versus DMSO controls; † *p* < 0.05 versus L-AA; ‡ *p* < 0.05 versus 35.1 μg/mL DCM. # *p* < 0.05 versus 35.1 μg/mL EA *n* = 3. (**b**) 30 min co-treatment of DCM and EA extracts inhibited 300 μM H_2_O_2_-induced ROS production in H9c2 cells. ROS levels were determined by DCFDA assay. * *p* < 0.05 versus H_2_O_2_ controls; † *p* < 0.05 versus DMSO controls; ‡ *p* < 0.05 versus NAC. *n* = 3. (**c**) Flow cytometry confirmed the anti-ROS effects from 30 min co-treatment of DCM and EA extracts with 300 μM H_2_O_2_ in H9c2 cells, and was followed by (**d**) quantitative analysis of DCF-positive population normalized to DMSO controls. * *p* < 0.05 versus H_2_O_2_ controls; † *p* < 0.05 versus DMSO controls. *n* = 3. (**e**) Representative fluorescent images visualized the anti-ROS effects of 30 min co-treatment of DCM and EA extracts with 300 μM H_2_O_2_ in NRCMs, which was followed by (**f**) quantitative analysis of probe intensity (DCF, green fluorescence). Scale bar = 500 μm. * *p* < 0.05 versus H_2_O_2_ controls; † *p* < 0.05 versus DMSO controls. *n* = 3. (**g**) Representative fluorescent images of mitochondrial superoxide after 30 min co-treatment of DCM and EA extracts with 300 μM H_2_O_2_ in NRCMs, which was followed by (**h**) quantitative analysis of probe intensity (MitoSOX, red fluorescence). Scale bar = 500 μm. * *p* < 0.05 versus H_2_O_2_ controls. *n* = 4. (**i**) Representative fluorescent images of intracellular iron treated with FeSO_4_ and (**j**) quantitative analysis of probe intensity (FerroOrange, red fluorescence). Scale bar = 500 μm. * *p* < 0.05 versus FeSO_4_; † *p* < 0.05 versus DMSO controls. ‡ *p* < 0.05 versus DCM. *n* = 5. (**k**) Quantitative analysis of ROS intensity after treatment with FeSO_4_. * *p* < 0.05 versus FeSO_4_. *n* = 5. Quantification of the cellular antioxidative enzyme levels of (**l**) catalase, (**m**) MDA, and (**n**) GPx and (**o**) GSH/GSSG ratio after 30 min co-treatment of DCM and EA extracts with 300 μM H_2_O_2_ in NRCMs. * *p* < 0.05 versus H_2_O_2_; † *p* < 0.05 versus DMSO controls; ‡ *p* < 0.05 versus EA. *n* = 3. Data obtained from three independent experiments (with *n* = 3 for each experiment) are represented by the mean ± SEM.

Given that H_2_O_2_ primarily induces the generation of ROS to instigate cell death and that recent research has highlighted the antioxidative properties of *ginnala* extracts, we delved into whether cellular levels of ROS would be mitigated following the application of DCM and EA extracts. To quantify and compare the ROS levels across various groups, we performed the DCFDA assay, a fluorescent indicator that detects oxidation under two models: hypoxia/reoxygenation (H/R) (Figure 2a) and H_2_O_2_-treated cells (Figure 2b). To validate these findings, we utilized N-Acetyl-L-cysteine (NAC) and L-ascorbic acid (L-AA), two established ROS scavengers, as positive controls. As a result, we observed that the DCM and EA extracts significantly restrained the ROS production in both models in a dose-dependent manner, starting from a concentration as low as 35.1 µg/mL (Figure 2a,b).

Notably, the DCM and EA extracts exhibited even more potent reductions in the total ROS levels compared to the NAC at a much higher concentration of 250 µg/mL. We also performed flow cytometry and obtained DCF images to assess the DCF signal, which indicated the ROS levels in each cell after treatment. Again, the DCM and EA extracts displayed stronger ROS scavenging effects than the NAC (Figure 2c–f). Moreover, we observed that the mitochondrial superoxide levels in the NRCMs were notably decreased post-extract treatment (Figure 2g,h), suggesting potential mitochondrial protective effects of DCM and EA extracts under oxidative stress. Lastly, a 1 h pre-treatment with *ginnala* extracts demonstrated similar ROS inhibitory effects (Figure S3), confirming the antioxidative properties of DCM and EA and their robust cardio-protection against cellular MIRI.

Given that ferroptosis is triggered by iron overload, we mixed the extracts with ferrous sulfate (FeSO_4_) and treated NRCMs. The FerroOrange assay demonstrated a substantial reduction in the intracellular Fe^2+^ levels in the groups that were treated with extracts (Figure 2i,j), indicating the potential protection of *ginnala* extracts against ferroptosis. Interestingly, the reduction in Fe^2+^ levels did not directly correlate with the decrease in ROS levels, as DCM showed relatively lower anti-iron effects but exhibited significantly higher anti-ROS efficacy (Figure 2j,k). The total ROS level in the DCM-treated NRCMs was even slightly lower than that in the DMSO controls, suggesting comprehensive antioxidative activity of *ginnala* extracts despite the lack of anti-ferroptosis effects.

Numerous antioxidants are known to enhance intracellular antioxidative enzymes to mitigate excessive ROS and safeguard cellular function under oxidative stress. To explore whether *ginnala* extracts could eliminate intracellular ROS by activating cellular enzymes, we evaluated the activities of widely reported antioxidative enzymes in NRCMs, including catalase (CAT), malondialdehyde (MDA), and glutathione peroxidase (GPx), as well as reduced glutathione (GSH) and oxidized glutathione (GSSG) (Figure 2l–o). However, although the CAT activity increased in the DCM-treated cells, no enhancements were observed in other antioxidant enzymes compared to the untreated controls. This suggests that *ginnala* extracts alleviate oxidative stress without directly promoting intracellular antioxidase activity.

### 3.3. Antioxidase-like Biocatalytic Performance of ginnala Extracts

**Figure 3:**
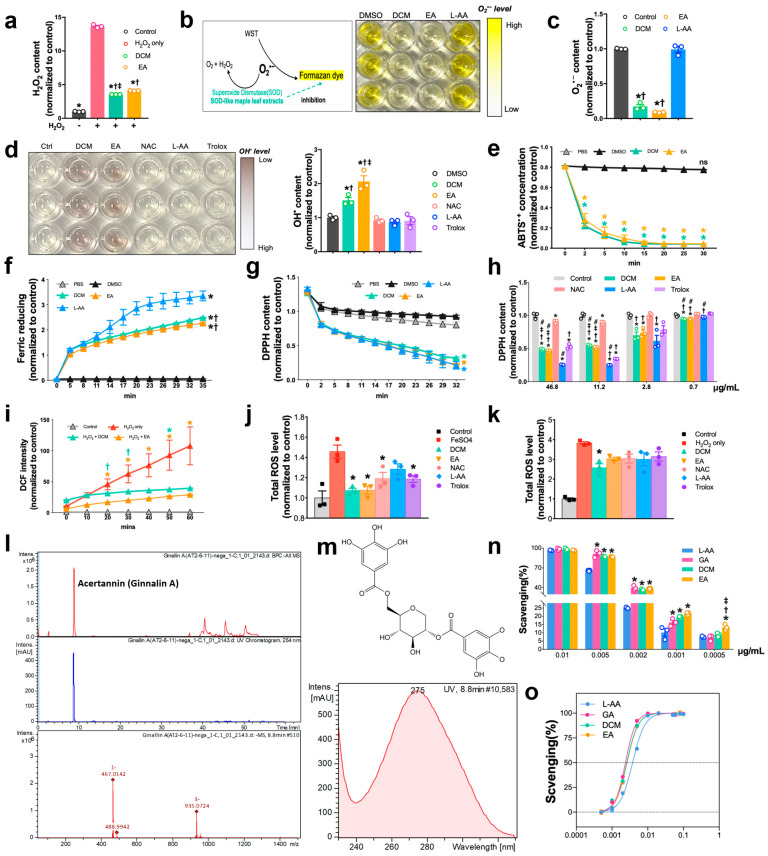
Antioxidase-like biocatalytic performance of *ginnala* leaf extracts. (**a**) Determination of H_2_O_2_ content after mixing with DCM and EA extracts. * *p* < 0.05 versus DMSO controls with 1 mM H_2_O_2_; † *p* < 0.05 versus DMSO controls. ‡ *p* < 0.05 versus EA. *n* = 3. (**b**) Scheme depicting the SOD-like activity of *ginnala* leaf extracts by comparing the remaining superoxide (O_2_^•−^) levels, followed by a photograph indicating the O_2_^•−^ levels obtained by using formazan dye. (**c**) Quantification analysis of O^2•−^ intensity. * *p* < 0.05 versus H_2_O_2_; † *p* < 0.05 versus L-AA. *n* = 3. (**d**) Photograph and quantification analysis of OH• radicals’ inhibition. * *p* < 0.05 versus DMSO controls; † *p* < 0.05 versus NAC, L-AA and Trolox. ‡ *p* < 0.05 versus DCM. *n* = 3. (**e**) Total antioxidant capacity of DCM and EA fractions measured by ABTS method. * *p* < 0.05 versus PBS controls; ns, not significant. *n* = 3. (**f**) Time-dependent Ferric clearance capacity of DCM and EA. L-AA was used as positive control. * *p* < 0.05 versus DMSO controls; † *p* < 0.05 versus L-AA. *n* = 3. (**g**) Time-dependent DPPH scavenging capacity of DCM and EA. * *p* < 0.05 versus DMSO controls. *n* = 3. (**h**) DPPH scavenging capacity of DCM, EA, and other ROS scavengers with low doses. * *p* < 0.05 versus DMSO controls; † *p* < 0.05 versus NAC; ‡ *p* < 0.05 versus L-AA; # *p* < 0.05 versus Trolox. *n* = 3. (**i**) Time-dependent ROS scavenging activities of DCM and EA extracts. * *p* < 0.05 versus H_2_O_2_; † *p* < 0.05 versus PBS controls. *n* = 4. (**j**) DCM and EA fractions lowered the intracellular ROS level induced by Fe^2+^. * *p* < 0.05 versus FeSO_4_. *n* = 3. (**k**) DCM and EA fractions lowered the intracellular ROS level induced by H_2_O_2_. * *p* < 0.05 versus H_2_O_2_. *n* = 3. (**l**) LC-MS Chromatogram of *Ginnalin A* from *Acer tataricum* L. subsp. *ginnala* (*Maxim.*) *Wesm.* leaves. (**m**) Chemical structure of *Ginnalin A*. (**n**) ABTS ^•+^ inhibitory effects of *ginnala* leaf extracts and L-AA at low doses. * *p* < 0.05 versus L-AA; † *p* < 0.05 versus GA; ‡ *p* < 0.05 versus DCM. *n* = 3. (**o**) Normalized ABTS^•+^ inhibition curve after treating DCM, EA, GA, and L-AA. *n* = 3. Data obtained from three independent experiments (with *n* = 3 for each experiment) are represented by the mean ± SEM.

Since the *ginnala* extracts exhibited strong anti-ROS effects without activating cellular antioxidative enzymes, we hypothesized that they function as a natural antioxidase, directly eliminating various environmental ROS to inhibit their production. To prove this hypothesis, we first investigated their CAT-like activities by mixing DCM and EA fractions with H_2_O_2_. Through the evaluation of the remaining H_2_O_2_ content, we observed significant H_2_O_2_ decomposition activities of the *ginnala* extracts (Figure 3a). The SOD-like activity (·O_2_^•−^ to H_2_O_2_) of DCM and EA fractions was also examined by determining the elimination ratio of ·O_2_^•−^ using the WST-1 formazan dye as a specific indicator (Figure 3b). Both the DCM and EA extracts displayed promising SOD-like activities by reducing the ·O_2_^•−^ levels and inhibiting the formation of the yellow formazan dye. In contrast, the common ROS scavenger L-AA did not exhibit any effects at the same dose of 46.8 µg/mL (Figure 3b,c). These results indicated a SOD–CAT catalytic ROS-scavenging activity of *ginnala* extracts against ·O_2_^•−^ and H_2_O_2_.

The hydroxyl radical (·OH) is a major source of intracellular oxidative stress as it is highly reactive and can readily react with various biological molecules. To evaluate the capability of *ginnala* extracts in clearing hydroxyl radicals, Fe^2+^ and H_2_O_2_ were utilized to create a Fenton system for hydroxyl radical generation. After the formation of hydroxyl radicals, DCM and EA fractions, along with the ROS scavengers NAC, L-AA, and Trolox at the same concentration (46.8 µg/mL), were introduced into the ·OH-containing solution. Ten seconds after administration, the color of the DCM- and EA-treated groups intensified compared to that of the untreated controls and the groups with other ROS scavengers, indicating an ultrafast ·OH elimination activity of *ginnala* extracts (Figure 3d).

Next, we assessed the total antioxidation capability of *ginnala* extracts by measuring the ABTS+ concentration of the samples. Upon co-incubation, the total antioxidation capacities of the DCM and EA fractions were determined to be significant, as demonstrated by the rapid ABTS+ reduction of approximately 70% at 2 min and the nearly 100% ABTS+ elimination at 20 min (Figure 3e). A ferric-reducing assay was also conducted to showcase the antioxidative capacity of *ginnala* extracts, with the scavenging activities of the DCM and EA fractions also manifesting within a short period (Figure 3f). Although there was a difference compared to the positive control (L-AA), it should be noted that the concentration of L-AA was 250 µg/mL, approximately five times higher than that of the extracts.

DPPH is a stable free radical with nitrogen as its center and was also employed to further investigate the antioxidative activities of *ginnala* extracts at multiple levels. The reduction in DPPH in the DCM and EA-administered groups was significant and rapid, with both eliminating approximately 70% of the DPPH within 20 min (Figure 3g). To explore the effects of low doses of extracts, DCM and EA extracts, along with other ROS scavengers, were diluted 4, 16, and 64 times (Figure 3h). The DPPH content was immediately determined (10 s) after mixing each group with DPPH to assess the rapidity of the resulting antioxidation. The scavenging effects remained promising even after 16- and 64-times dilution of the candidates, while NAC, believed to activate cellular enzymes, elicited no change in the DPPH levels after mixing. Starting at a concentration of 11.2 µg/mL, *ginnala* extracts displayed superior radical scavenging effects at low concentrations, while L-AA and NAC required a higher concentration to eliminate DPPH (Figures S4 and S5).

After confirming the scavenging activities of the studied extracts against different ROS-inducers, we used the DCFDA assay to determine the total ROS levels in the reaction environment. Although DCFDA is primarily effective with live cells, as intracellular esterase is needed to produce a fluorescent compound after oxidation, it is known that the base-catalyzed hydrolysis of esters can occur without esterase, and H_2_O_2_, which provides one-electron-oxidizing species, can oxidize DCFH to DCF. Therefore, we directly mixed DCFDA with a high concentration of H_2_O_2_ (10 mM), and found that, in this mixture, the DCF signals were notably higher than in the PBS controls. However, 20 min after the DCM and EA fractions were added, the DCF signals decreased rapidly (Figure 3i). Furthermore, the total ROS levels were reduced by the DCM and EA fractions in a dose-dependent manner (Figure S6). Surprisingly, the best ROS scavenging effects were achieved at a concentration of 5.7 µg/mL of the DCM and EA fractions, indicating optimal efficacy at a lower concentration. Remarkably, the H_2_O_2_ concentration used in this assay was 20 times higher (10 mM) than that in the cell viability assay (500 µM), suggesting that the *ginnala* extracts not only protected cells against extracellular ROS but also improved the intracellular condition after H_2_O_2_ exposure. This finding was consistent with the observation of an energetic state in NRCMs after 30 min of extract treatment.

To further demonstrate the rapid anti-ROS effects of these extracts in cells, we treated NRCMs with two ROS-inducers, H_2_O_2_ and FeSO_4_, to induce intracellular oxidative stress, which was indicated by the DCF intensity. After observing an increase in intracellular ROS compared to untreated controls, we removed H_2_O_2_ and FeSO_4_ from the NRCMs by rinsing them with PBS and then added the DCM and EA fractions and other ROS scavengers at the same dose (46.8 µg/mL) to the stressed cells. For the FeSO_4_-treated cells, all ROS scavengers, except L-AA, significantly inhibited ROS after 15 min (Figure 3j). In the case of the H_2_O_2_-treated cells, although all five scavengers (including NAC) reduced the ROS levels, only the DCM fraction exhibited significant protective effects at 15 min (Figure 3k). Monitoring the decline in ROS levels in each group revealed a clear decrease 1 min after adding the DCM fractions, although it was not statistically significant for any of them (Figure S7a). After extending the incubation time to 30 and 150 min for each group, except for NAC, the inhibition was sustained by the scavengers (Figure S7b), which indicated a significantly long-term ROS regulatory effect. These results collectively demonstrate that *ginnala* extracts exhibited antioxidase-like biocatalytic performance and rapidly and robustly eliminated various ROS-inducers.

### 3.4. The Primary Active Component in ginnala Extracts Is Ginnalin A

We confirmed that *Ginnalin A* (>98% purity, as determined by HPLC-MS) was the major compound in the leaves of *Acer tataricum* L. subsp. *ginnala* (*Maxim.*) *Wesm*. It appeared as a white amorphous powder with a chemical formula of C20H20O13 and a molecular weight of 468 g/mol. The 1H-NMR and 13C-NMR spectra data were consistent with previous reports, confirming the identity of the *Ginnalin A* [12] (Figure 3l). The chemical structure of *Ginnalin A*, shown in Figure 3m, features two 3,4,5-trihydroxybenzoic acid moieties at both ends, making it the strongest phenolic acid in scavenging various radical compounds. Additionally, the symmetry of *Ginnalin A* displayed superior stability, which enabled it to be more stable than other antioxidants with asymmetric structures, such as ascorbic acid, Trolox, gallic acid, catalase, and superoxide dismutase (Figure S8), whose antioxidative capacity is easily impaired due to degradation. To further validate the potent antioxidant profile of *ginnala* extracts and commercially obtained *Ginnalin A*, we used an ABTS assay to measure their ABTS radical scavenging effects. Concentrations ranging from 0.0005 to 0.1 mg/mL of the extracts and *Ginnalin A* were mixed with an ABTS+ solution, and the remaining ABTS+ was detected and visualized by the intensity of the green color immediately after the reaction (10 s) (Figure 3n,o and Figure S9). The results showed that, at low concentrations ranging from 0.005 to 0.0005 mg/mL, the DCM and EA fractions, along with *Ginnalin A*, displayed powerful ABTS+ scavenging activities with equivalent and even lower IC50 values compared with L-ascorbic acid. To confirm that *Ginnalin A* could re-produce the observed protective effects seen in DCM and EA extracts, we co-treated *Ginnalin A* with H_2_O_2_ on NRCMs, and, as expecteded, *Ginnalin A* sustained the observed efficacy in salvaging viable cells. (Figure S10).

### 3.5. ginnala Extracts Prevented Erastin and RSL3-Induced Ferroptosis

**Figure 4:**
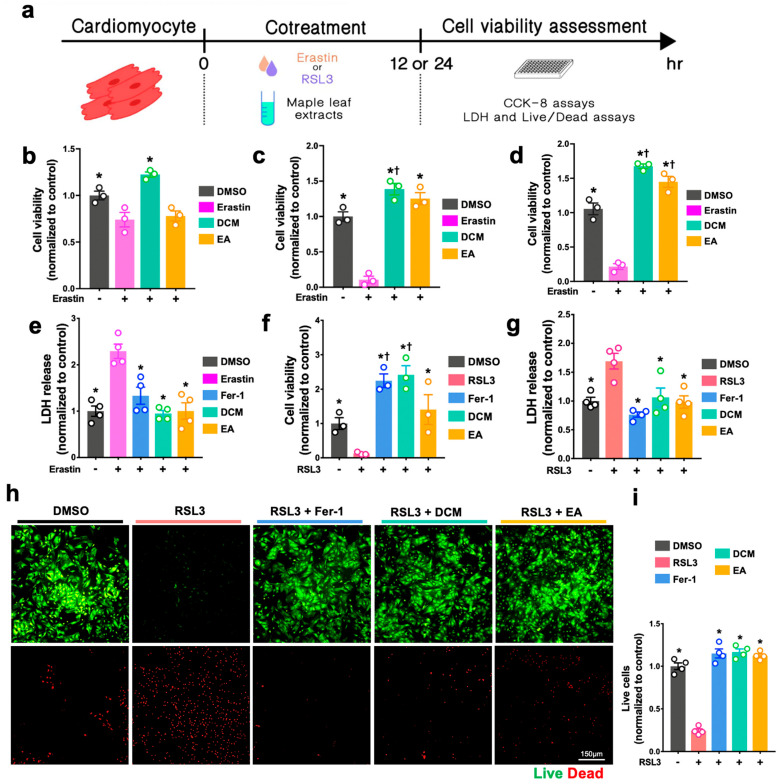
*ginnala* leaf extracts prevented Erastin- and RSL3-induced cellular ferroptosis. (**a**) Experimental protocol for in vitro ferroptosis injury: 12 or 24 h incubation of extracts along with 35 μM Erastin or 2 μM RSL3. (**b**,**c**) *ginnala* leaf extract candidates exhibited cytoprotective effects in 12 h and 24 h co-treatment with Erastin on H9c2 cardiomyoblast as determined by CCK-assay. (**d**) 24 h co-treatment of extracts with Erastin on NRCMs and (**e**) NRCMs injury was evaluated by extracellular release of LDH. (**f**,**g**) Cell viability and LDH release were also assessed in NRCMs after 24 h co-treatment with RSL3. (**h**,**i**) NRCMs viability was further evaluated by live/dead staining: Representative images and quantification summary of both live and dead cells per field. * p < 0.05 versus Erastin- or RSL3-treated controls. † *p* < 0.05 versus DMSO controls. *n* ≥ 3. Scale bar = 150 μm. Data obtained from three independent experiments (with *n* = 3 for each experiment) are represented by the mean ± SEM.

Given that ferroptosis has been reported as a novel form of cell death, which can also be induced by lipid peroxidation with excessive ROS during IR injury [13], we further tested if DCM and EA fractions can also protect cardiomyocytes from ferroptotic cell injury and therefore confer comprehensive protection against MIRI. Rather than the acute cell injury induced by H_2_O_2_, the occurrence of ferroptosis requires a longer action time of the ferroptosis inducer. Thus, with longer incubation times of 12 and 24 h, we first ascertained the appropriate concentration of two ferroptosis inducers, RSL 3 and Erastin, for triggering cell death in H9c2 cells and NRCMs. We found that ~35 µM of Erastin induced ~50% cell death while RSL3 induced similar damage at a dose of ~2 µM (Figure S11). When using the same co-treatment protocol but a longer duration and incubating cells using the DCM and EA fractions together with Erastin or RSL3 (Figure 4a), CCK-8 assays firstly demonstrated a robust cardiac protective effective of the extracts that was due to them significantly improving the H9c2 cell viabilities (Figure 4b,c). The anti-ferroptosis effects of DCM and EA on Erastin- and RSL3-treated NRCMs were also significant, with a much higher number of remaining cells (Figure 4d,f) and less LDH release (Figure 4e,g), and were comparable with the effects of ferrostatin-1 (fer-1), a specific inhibitor of ferroptosis [14]. These results were further evidenced using live/dead analysis, in which a larger population of live cells were found in the DCM and EA extract-treated NRCMs (Figure 4h,i). The observed anti-ferroptosis effects suggested long-term protective roles of *ginnala* extracts. Given that long-term administration could easily produce side effects, to assess the toxicity of DCM and EA fractions, both low and high concentrations of *ginnala* extracts were incubated in NRCMs for 3 days. Surprisingly, the LDH assay suggested less LDH release in the DCM- and EA-treated NRCMs. More interestingly, the high concentration of EA extracts (128.7 µg/mL) induced minimal cellular damage compared with untreated cells (Figure S12).

### 3.6. ginnala Extracts Promoted an Energetic Cell State of Cardiomyocytes

During MIRI, there is a swift recovery of fatty acid oxidation but a decline in the rate of glycolysis oxidation, indicating that enhancing glycolysis could be beneficial in MIRI therapeutics. Enhanced glycolysis has been linked to cardioprotection in MIRI, and considering the anti-glycemia role of *ginnala* extracts, we posited that the administration of DCM and EA fractions could stimulate glycolysis in NRCMs and confer cardiac protective effects.

**Figure 5:**
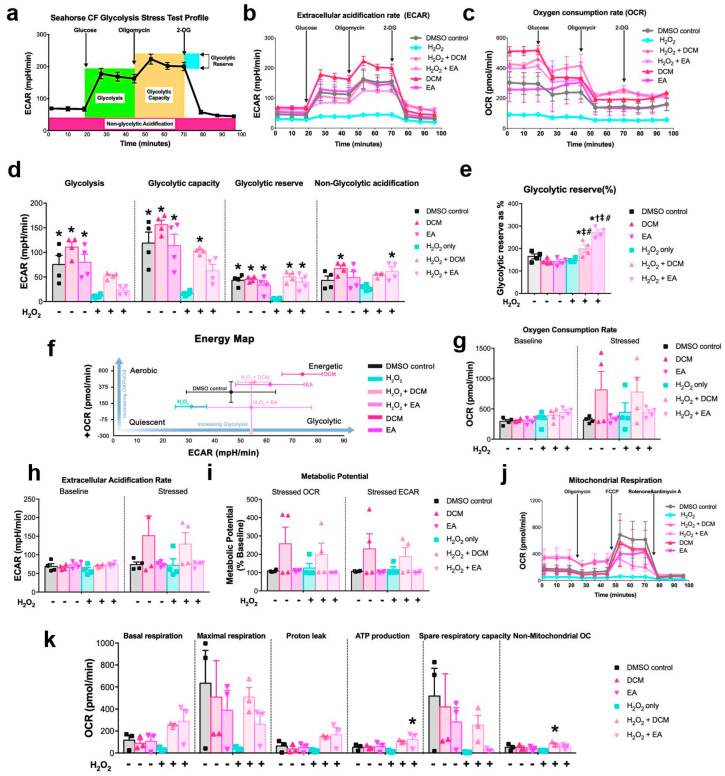
*ginnala* leaf extracts promoted an energetic cell state of cardiomyocytes. (**a**) Graphical representation of glycolytic stress (ECAR) profile. (**b**) Measurement of extracellular acidification rates (ECAR) in NRCMs after 30 min co-treatment of DCM and EA extracts with 300 μM H_2_O_2_ by glycolysis stress assay. *n* = 3. (**c**) Measurement of oxygen consumption rate (OCR) by glycolysis stress assay. *n* = 3. (**d**) Quantification of glycolysis, glycolytic capacity, glycolytic reserve, and non-glycolytic acidification. * *p* < 0.05 versus H_2_O_2_ controls. *n* = 3. (**e**) Quantification of percentage of glycolytic reserve from each group. * *p* < 0.05 versus H_2_O_2_ controls; † *p* < 0.05 versus DMSO controls; ‡ *p* < 0.05 versus DCM without H_2_O_2_; # *p* < 0.05 versus EA without H_2_O_2_. *n* = 3. (**f**) Energy profile of NRCMs generated from this glycolysis measurement. *n* = 3. (**g**–**i**) OCR, ECAR, and metabolic potential of NRCMs from baseline and stressed conditions. *n* = 3. (**j**) Measurement of oxygen consumption rate (OCR) in NRCMs after 30 min co-treatment of DCM and EA extracts with 300 μM H_2_O_2_ by mitochondrial stress assay. *n* = 3. (**k**) Quantification of basal respiration, maximal respiration, proton leak, ATP production, and non-mitochondrial oxygen consumption. * *p* < 0.05 versus H_2_O_2_ controls. *n* = 3. Data represent the mean ± SEM.

Following the co-treatment protocol against H_2_O_2_, we monitored the metabolic dynamics of NRCMs using the seahorse glycolysis stress assay 30 min after treatment with DCM and EA fractions in the presence or absence of H_2_O_2_. Notably, the H_2_O_2_ concentration used was halved to prevent acute cell death. The real-time extracellular acidification rate (ECAR) and oxygen consumption rate (OCR) decreased 30 min after exposure to H_2_O_2_, while treatment with DCM and EA extracts restored the ECAR and OCR (Figure 5a–c). Specifically, the glycolysis, glycolytic capacity, reserve, and non-glycolytic acidification were enhanced after the administration of DCM and EA (Figure 5d). Although the increase was subtle compared to untreated controls, there was a significant improvement in the percentage of glycolytic reserve in the DCM- and EA-treated NRCMs in the presence of H_2_O_2_ (Figure 5e), indicating that the *ginnala* extracts stimulated a rapid cellular response to energetic demand under oxidative stress. The cell energy map illustrated that the DCM and EA fractions significantly bolstered aerobic and glycolytic respiration of the NRCMs, highlighting that an energetic state was induced irrespective of the presence of H_2_O_2_ (Figure 5f). Another assay targeting the cell energy phenotype revealed that the DCM fractions elevated the NRCMs into a more energetic state in which the OCR and ECAR were improved and the metabolic potential generated by the difference between the OCR and ECAR was also increased (Figure 5g–i). Since MIRI has been linked to the loss of cell energy, the enhancement of the cell energy phenotype by *ginnala* extracts could signify cardioprotection against MIRI.

During MIRI, excessive ROS can impair mitochondrial function and hamper ATP production. Thus, we investigated whether *ginnala* extracts could positively impact mitochondrial function by enhancing cellular energy production. Through a mitochondrial stress assay to monitor the oxygen consumption rate (OCR) in NRCMs treated with various modulators, we noted a significant enhancement in mitochondrial function and ATP production upon the inclusion of H_2_O_2_ (Figure 5j,k). While there were improvements in other parameters, such as the basal respiration, none were statistically significant.

Moreover, we also found that, after treatment with DCM and EA extracts for a longer time (3 days), the NRCM cell size was much smaller than DMSO-treated controls (Figure S13), which suggested the potential roles of *ginnala* extracts in preventing cardiomyocyte hypertrophy. It is known that hypertrophic growth features in the cellular senescence of cardiomyocytes and aged cells also have a decreased energy consumption compared with young and well-beating cells. In this regard, we postulate that *ginnala* extracts restore the energetic state of isolated cardiomyocytes and thereby prevent their hypertrophy and senescence.

### 3.7. In Vivo Cardioprotective Effect of ginnala Extracts After Ischemic-Reperfusion Injury

To verify the antioxidant properties of ginnala extracts in MIRI, intramyocardial injections of EA or DCM fractions were administered 5 min before reperfusion. Evans blue and TTC staining were conducted 24 h post-IR injury.

**Figure 6:**
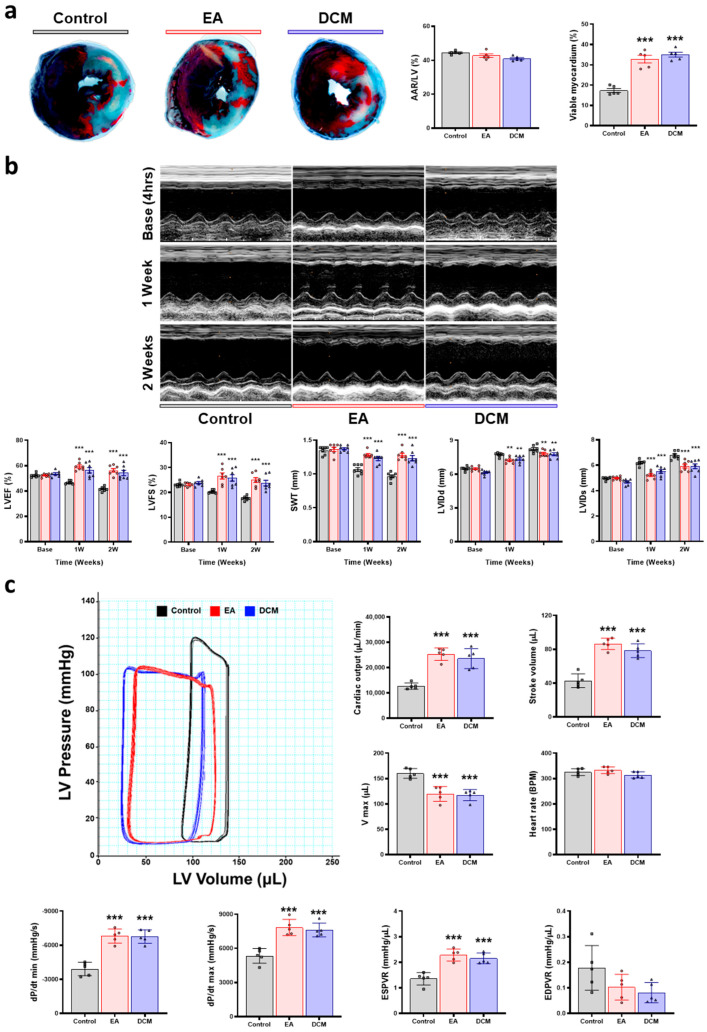
Early cardiac protective effect and cardiac function preservation effect of the *ginnala* leaf extracts after ischemic-reperfusion injury. (**a**) Representative images of Evans blued and TTC staining at 24 h after inducing ischemic-reperfusion injury and quantification of area at risk (AAR)/LV and viable myocardium at risk area. *n* = 5. *** *p* < 0.001 vs. control. Data are shown as the mean  ±  S.E.M. (**b**) Representative images of M-mode of three groups at base (4 h), 1 week, and 2 weeks after injury. The quantification of the left ventricular ejection fraction (EF), left fractional shortening (FS), septal wall thickness (SWT), left ventricular internal dimension at end-diastole (LVIDd), left ventricular internal dimension at end-systole (LVIDs) was conducted. *n* = 7. ** *p* < 0.01 vs. control. *** *p* < 0.001 vs. control. Data are shown as the mean  ±  S.E.M. (**c**) Representative images of the hemodynamic pressure and volume (PV) curve of steady state at 2 weeks post-IR injury. Quantification data of cardiac output, stroke volume, volume max (V max), heart rate, maximal rate of pressure changes during systole (dP/dtmax), minimal rate of pressure changes during diastole (dP/dtmin), slope of end-systolic pressure–volume relationship (ESPVR), slope of end-diastolic pressure–volume relationship (EDPVR). *n* = 5. ***p < 0.001 vs. control. Data are shown as the mean  ±  S.E.M. Statistical significance was determined using unpaired two-sided Student’s *t*-tests in (**b**) and one-way ANOVA with Tukey’s multiple comparisons tests in (**a**,**c**).

While there was no disparity in the area at risk within the left ventricular (LV) wall (Evans blue-negative area), the viable myocardium within the risk area (Evans blue-negative and TTC staining-positive area) exhibited a significant increase in the EA and DCM groups compared to the control group (Figure 6a). This outcome suggests that *ginnala* extracts offer an early defense mechanism against IR injury.

To evaluate whether this initial protective effect positively influenced cardiac function, serial echocardiography was performed at baseline (4 h), 1 week, and 2 weeks post-MIRI. Although there were no discernible differences in cardiac function among the groups during the baseline echocardiography, a significant protective impact on cardiac function was noticeable starting from week 1 and sustained through the second week. The left ventricular ejection fraction (LVEF) and left ventricular fractional shortening (LVFS) showed marked enhancements in the EA and DCM groups compared to the control group, with the septal wall thickness (SWT) being preserved in the EA and DCM groups. Furthermore, the left ventricular internal dimension at the end-diastole (LVIDd) and left ventricular internal dimension at the end-systole (LVIDs) were smaller than in the control group (Figure 6b).

The hemodynamic cardiac function was evaluated through pressure–volume (PV) loop testing after 2 weeks of ischemic-reperfusion injury. Both the stroke volume and cardiac output displayed improvements in the EA and DCM groups, with the maximum volume (V max) being lower than in the control group. Comparable heart rates were recorded across all groups. Additionally, the maximum rate of pressure change (dP/dt max) and the minimum rate of pressure change (dP/dt min) notably increased in the EA and DCM groups. The load-independent cardiac contractility, induced by transient occlusion of the inferior vena cava, revealed that the EA and DCM groups exhibited superior contractile function compared to the control group, including a steeper slope of the end-systolic pressure–volume relationship (ESPVR). The slope of the end-diastolic pressure–volume relationship (EDPVR) was consistent across all groups (Figure 6c and Figure S14). Parameters such as the LVEF, FS, and dP/dt showed marked improvements at both 1 and 2 weeks post-I/R injury. In several cases, the functional values are levels that are typically reported for sham animals in similar models, although this cannot be formally confirmed without a sham group. The degree of recovery observed in the EA- and DCM-treated groups approached the levels commonly reported in sham-operated animals in similar experimental settings, suggesting that the ginnala extracts provided robust cardioprotection, likely restoring cardiac function to near-normal levels despite the absence of direct sham comparison.

**Figure 7:**
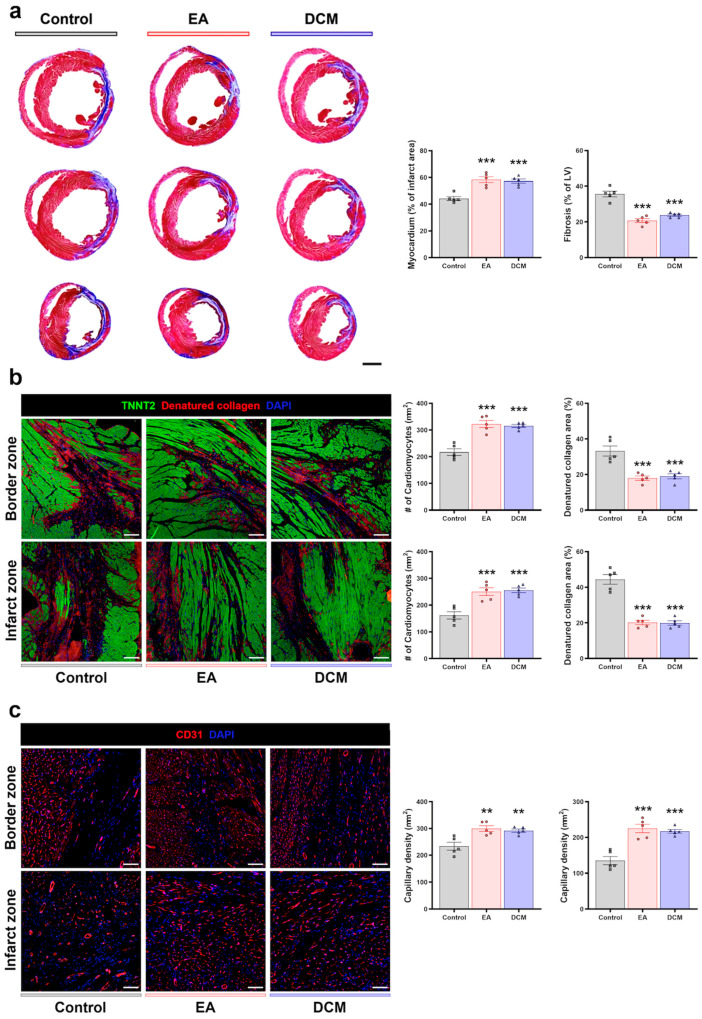
*ginnala* leaf extracts improved cardiac microenvironment after ischemic-reperfusion injury. (**a**) Representative images of Masson’s trichrome staining at 2 weeks and quantification summary of a percentage of fibrosis and myocardium. *n* = 5. *** *p* < 0.001 vs. control. Scale bars: 2000 µm. Data are shown as the mean  ±  S.E.M. (**b**) Representative images of cardiomyocytes stained with cardiac troponin T (TNNT2) (green) and denatured collagen (red) on the infarct zone, border zone at 2 weeks, and their quantification summary (#, number of cardiomyocytes per mm²). *n* = 5. *** *p* < 0.001 vs. control. Scale bars: 200 µm. Data are shown as the mean  ±  S.E.M. (**c**) Representative images of capillaries stained with CD31 (red) on the infarct zone, border zone at 2 weeks, and their quantification summary. *n* = 5. ** *p* < 0.01 vs. control. *** *p* < 0.001 vs. control. Scale bars: 200 µm. Data are shown as the mean  ±  S.E.M. Statistical significance was determined using one-way ANOVA with Tukey’s multiple comparisons tests.

Histological examination was also performed at the 2 weeks post-IR injury to compare the level of fibrosis in the heart using Masson’s trichrome staining. The assessment unveiled a decrease in the fibrotic area (aniline blue-positive area) in the EA and DCM groups in comparison to the control group. Moreover, the myocardial tissue (scarlet red-positive area) within the infarct site, where fibrosis had accumulated, exhibited an augmentation relative to the control group (Figure 7a). Immunohistochemical staining for cardiac troponin T (TNNT2) and denatured collagen was carried out, and indicated improved survival of cardiomyocytes and a reduction in denatured collagen deposition in both the border zone and infarct zone (Figure 7b). Analysis of the capillaries through CD31 immunohistochemical staining demonstrated a higher quantity of capillaries in the border zone and infarct zone in the EA and DCM groups compared to the control group (Figure 7c).

From these findings, it can be inferred that the ROS scavenging properties of EA and DCM fractions protected cardiomyocytes against MIRI, resulting in improved cardiac function. Additionally, our findings indicate that *ginnala* leaves improved the cardiac microenvironment by reducing fibrotic deposition, enhancing cardiomyocyte survival, and increasing capillary presence, which are likely attributable to their early protective effects.

## 4. Discussion

Although several antioxidants, including recombinant human SOD, have undergone preliminary clinical trials for their potential therapeutic roles in eliminating ROS and managing MIRI, these treatments have demonstrated limited efficacy in improving heart function recovery in patients undergoing coronary angioplasty for myocardial ischemia. This limited effectiveness stems from the sudden surge in oxygen levels and the restricted capacity of these drugs to regulate oxidative stress. ROS, triggered by the surge in oxygen upon blood flow restoration, is the primary culprit behind MIRI. Hence, it is imperative to identify potent ROS scavengers that are capable of effectively mitigating MIRI.

In the present study, we aimed to pinpoint a natural antioxidase known as *Ginnalin A*, which is sourced from the leaves of *Acer tataricum* L. subsp. *ginnala* (*Maxim.*) *Wesm*, a variety of amur maple trees. By subjecting various fractions with different concentrations to cardiomyocytes under oxidative stress, we showcased the swift and comprehensive ROS scavenging attributes of *Ginnalin A*. It efficiently neutralizes diverse ROS and free radicals, including O_2_^−^, H_2_O_2_, OH•, and Fe^2+^, and thus delivers prompt and robust cardioprotection when introduced into both in vitro and in vivo models.

Contrary to the usual protocol of administering drugs before percutaneous coronary intervention (PCI), our study primarily employed a co-administration method, where *ginnala* extracts were concomitantly administered with ROS inducers. This approach aimed to closely replicate the clinical urgency during heart reperfusion. Within a concise 30 min window, the *ginnala* extracts exhibited ultrafast suppression of ROS production and safeguarded cardiomyocyte viability. Through a series of assays assessing antioxidative prowess, we observed that the *ginnala* extracts demonstrated rapid and sequential ROS scavenging characteristics. These extracts showcased behaviors akin to SOD and CAT within minutes. Notably, they effectively targeted a broad spectrum of ROS species, including O_2_^•−^ and OH•, outperforming common antioxidants like ascorbic acid, NAC, and Trolox, even at equivalent concentrations. Furthermore, the *ginnala* extracts significantly shielded cardiomyocytes against oxidative harm induced by H_2_O_2_ and ferroptosis, which underscores their potential to salvage impaired myocardium, fortify vasculature, and enhance cardiac functions. To delve deeper into the antioxidative attributes of these extracts, we conducted a comparative analysis with three conventional antioxidants—ascorbic acid, NAC, and Trolox. Various ROS species were employed to evaluate their scavenging efficacy. While ascorbic acid exhibited superior effects at higher concentrations, when tested at identical doses to the extracts, only the effects of the *ginnala* extracts persisted rapidly. Leveraging the ABTS assay, which reflects of anti-radical activity by gauging the remaining ABTS^•+^ radicals, we determined that the *ginnala* extracts exhibited superior scavenging effects with a lower IC50 in comparison to ascorbic acid. Taken together, these results suggest that *ginnala* extracts confer ultrafast and cascade-like biocatalytic activities through chemically derived functional groups that effectively mimic intracellular ROS defense mechanisms, such as those of SOD and CAT. Unlike regulatory effects that involve modulating molecular pathways within cells, these catalytic actions enable ginnala extracts to serve as a timely and effective approach for sensing and neutralizing bursts of ROS, and their quick action suits the clinical urgency encountered during myocardial IR injury.

The antioxidative effects of *ginnala* extracts transcend their capacity to scavenge extracellular ROS from the environment. The direct application of the extracts on NRCMs under normoxic conditions resulted in a reduction in cellular ROS levels, indicating a proactive effect upon cellular entry. Rather than eliciting cellular antioxidative enzymes such as SOD, CAT, and GPx, the extracts embedded themselves within the cells, acting as potent antioxidases that efficiently regulated cellular oxidative stress. Consequently, when NRCMs were exposed to the ferroptosis inducers RSL3 and Erastin, which typically necessitate prolonged incubation times for their effects to manifest, for 12 and 24 h, the protection conferred by the *ginnala* extracts remained significant. This underscores the comprehensive and sustained rapid ROS-eliminating effects of the studied extracts.

Mass spectrometry analysis unveiled *Ginnalin A* (GA) as a pivotal polyphenolic component that was present in the *ginnala* extracts, which aligns with prior reports. GA, characterized by two gallic acid groups situated at each end of the molecule, is recognized for its robust antioxidant activity compared to pyrogallol. The chemical structure of GA, a galloyl ester derivative of 3,4,5-trihydroxybenzoic acid, has exhibited the highest efficacy in inhibiting lipid peroxidation, scavenging hydrogen peroxide, and neutralizing DPPH radicals, surpassing other phenolic acids. One standout advantage of natural antioxidants like phenolic acids is their therapeutic efficacy coupled with their favorable safety profile. In assessing the toxicity of the *ginnala* extracts, we extended the treatment duration to three days and observed that the release of lactate dehydrogenase (LDH) from cells treated with the extracts was comparable to or even lower than that of the control group, highlighting the excellent biosafety of these natural extracts. Furthermore, by assessing the size of cardiomyocytes, we demonstrated the anti-hypertrophy potential of the extracts, which hints at long-term beneficial effects in heart repair in addition to their immediate protective attributes. Interestingly, 30 min treatment with the extracts induced an energetic state in NRCMs, with the extracts stimulating increased glycolysis and mitochondrial respiration. Furthermore, hemodynamic pressure and volume (PV) measurements showcased a substantial enhancement in cardiac contractility post the administration of the *ginnala* extracts, signifying their capability to sustain myocardial energy levels during MIRI.

Despite these promising results, there are some limitations that require further efforts to achieve the widespread therapeutic application of *ginnala* extracts. Given that myocardial IR injury impairs different kinds of cells in the heart, the selection of cardiomyocytes as the major target during heart therapy could disregard the indispensable contributions from other cell types, including endothelial cells, immune cells, and cardiac fibroblasts. For instance, although the in vivo results demonstrated higher density of the capillary, it could be more significant to investigate and confirm the direct effects of *ginnala* extracts on the cellular behavior of endothelial cells, as they could potentially assist in angiogenesis. In addition, intramyocardial injection, while offering precise delivery, could induce unneglectable damage to the myocardium due to mechanical invasiveness. Thereby, identifying other approaches such as intrapericardial administration and epicardial patch implantation could greatly prevent undesired risk and allow the sustained release of *ginnala* extracts to achieve more effective and safe treatment. Thirdly, regarding the long-term benefits of *ginnala* extracts in limiting cardiac hypertrophy, a comprehensive transcriptomic analysis of extracts treated with cardiomyocytes and myocardia is essential to further unravel the underlying mechanism that confers clinical relevance to the generation *ginnala* extract-based heart care products as precautions against hypertrophic cardiomyopathy (HCM).

## 5. Conclusions

In summary, our current study provides compelling evidence for the effectiveness of *ginnala* extracts as rapid and all-encompassing natural antioxidases. They efficiently neutralize excessive ROS within a short duration, furnishing timely protection to the heart during MIRI. By concentrating on the potent ROS scavenging activities of these extracts, we showcased their robust cardioprotective effects during reperfusion injury. Additionally, the identification of *Ginnalin A* highlights the potential of exploring the therapeutic effects of *ginnala* extracts on other ROS-related diseases. Overall, this study contributes to our understanding of the cardioprotective properties of *ginnala* extracts and paves the way for further research into their applications in various pathological conditions associated with oxidative stress.

## Data Availability

The data presented in this study are available in article and Supplementary Materials.

## Supplementary Materials

The following supporting information can be downloaded at: https://www.mdpi.com/article/10.3390/antiox14060671/s1, Figure S1: Flowchart of separation from leaves on *Acer tataricum* L. subsp. *ginnala* (*Maxim.*) *Wesm.*; Figure S2: 1 h Pre-treatment of *ginnala* leaf extracts alleviated H_2_O_2_-induced cell death.; Figure S3: 1 h Pre-treatment of *ginnala* leaf extracts inhibited H_2_O_2_-induced oxidative stress; Figure S4: Dose-dependent DPPH scavenging activities of *ginnala* leaf extracts and other common ROS scavengers; Figure S5: Time-dependent DPPH scavenging activities of *ginnala* leaf extracts and other common ROS scavengers; Figure S6: ROS scavenging activities from ginnala leaf extracts were dose dependent; Figure S7: *ginnala* leaf extracts exhibited faster intracellular ROS scavenging at same concentration of 46.8 μg/mL; Figure S8: Chemical structures of *Ginnalin A* and other common ROS scavengers.; Figure S9: ABTS^•+^ scavenging effects from *ginnala* leaf extracts and L-AA at 5 and 10 min; Figure S10: *Ginnalin A*(GA) protected NRCMs against H_2_O_2_-induced cellular damage; Figure S11: Screening of Erastin and RSL3 from different concentration for inducing cell death of NRCMs; Figure S12: Cellular toxicity evaluation of *ginnala* leaf extracts; Figure S13: *ginnala* leaf extracts prevented cardiomyocytes hypertrophy; Figure S14: Representative images of the slope of end-systolic pressure volume relationship (ESPVR).

## Abbreviations

The following abbreviations are used in this manuscript:

NRCMs	neonatal rat cardiomyocytes
LDH	lactase dehydrogenase
DPPH	2,2-diphenyl-1-picrylhydrazyl
ROS	reactive oxygen species
DCM	dichloromethane
EA	ethyl acetate
CAD	coronary artery disease
MI	myocardial infarction
MIRI	myocardial ischemia/reperfusion injury
SOD	superoxide dismutase
DCFDA	2′,7′-Dichlorofluorescin Diacetate
EF	ejection fraction
FS	fractional shortening
HX	hexane
H/R	hypoxia/reoxygenation
L-AA	L-ascorbic acid
NAC	N-Acetyl-L-cysteine
CAT	catalase
MDA	malondialdehyde
GPx	glutathione peroxidase
SWT	septal wall thickness
LVIDd	left ventricular internal dimension at end-diastole
LVIDs	left ventricular internal dimension at end-systole
ESPVR	end-systolic pressure–volume relationship
EDPVR	end-diastolic pressure–volume relationship
