# Supplementary material for: Ultrafast ROS Scavenging Activity of Amur Maple Tree Extracts Confers Robust Cardioprotection for Myocardial Ischemia/Reperfusion Injury
Source: Antioxidants (Basel). 2025 May 31;14(6):671. doi: 10.3390/antiox14060671 (PMC12189229; doi:10.3390/antiox14060671)
Supplement: Supplementary file 1 [file antioxidants-14-00671-s001.zip › antioxidants-3595804-supplementary.pdf]

Supplementary Materials for

Ultrafast ROS scavenging activity of amur maple tree extracts confers robust cardioprotection for myocardial ischemia/reperfusion injury

Aoyang Pu *et al.*

*Corresponding author: ban.kw@cityu.edu.hk; cardioman@catholic.ac.kr; leekt99@korea.kr

This PDF file includes:

Figs. S1 to S14

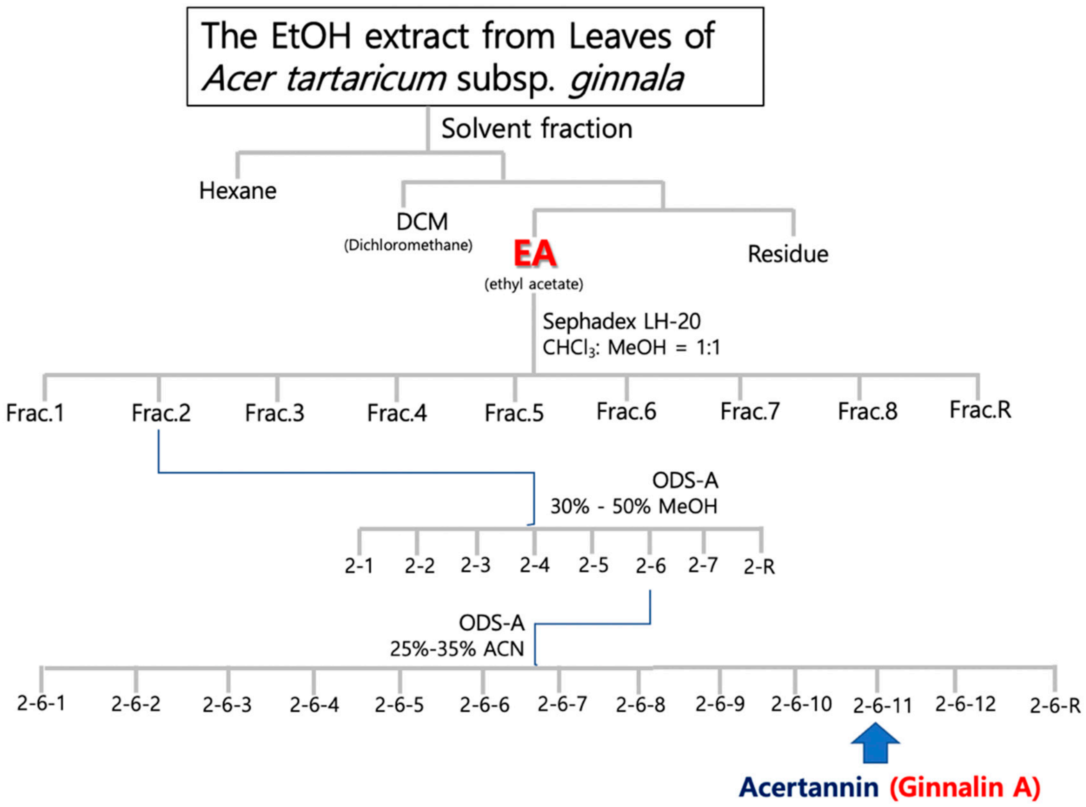

Supplementary Figure S1. Flowchart of separation from leaves on *Acer tataricum* L. subsp. *ginnala (Maxim.) Wesm.*

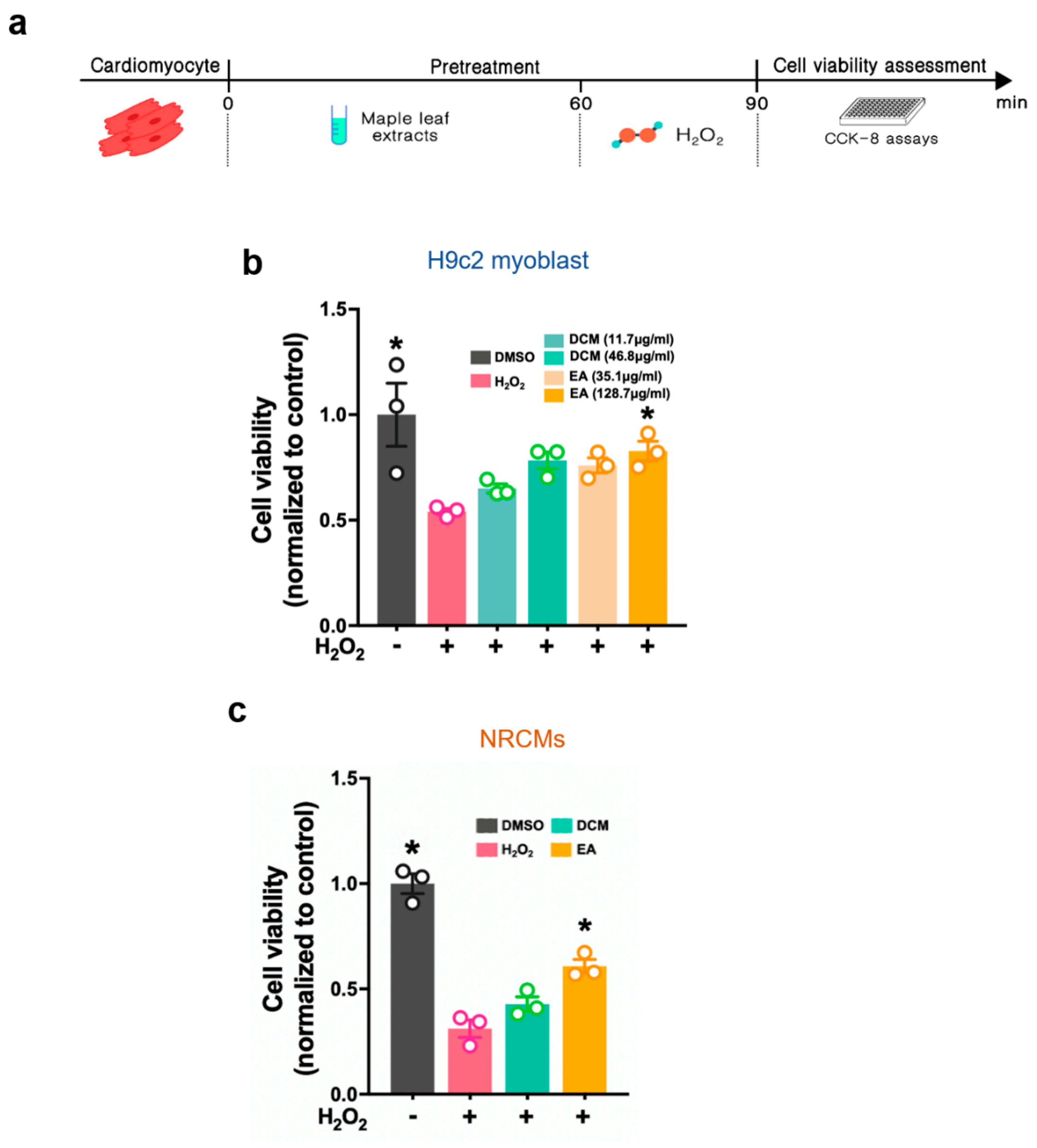

Supplementary Figure S2. 1hr Pre-treatment of *ginnala* leaf extracts alleviated H_2O_2-induced cell death. (**a**) Pre-treatment protocol demonstrates 1hr incubation of extracts prior to 800μM H_2O_2 treatment. **(b)** H9c2 viability was measured by CCK-8 assay after 1hr pretreatment of DCM and EA prior to 30min injury induced by H_2O_2. **(c)** The similar protective effect was also validated in NRCMs by employing the optimal concertation of DCM (46.8μg/mL) and EA 128.7μg/mL). * $p < 0.05$ versus H_2O_2. n=3. Data represent the mean ± SEM.

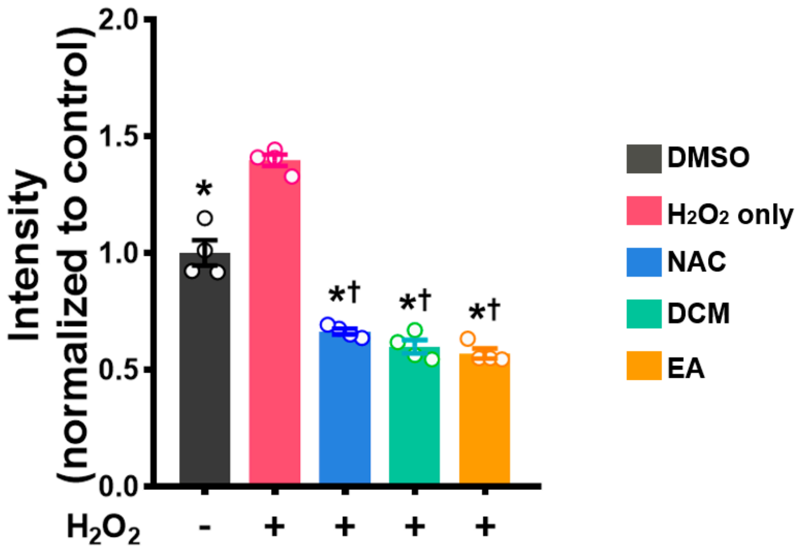

Supplementary Figure S3. 1hr Pre-treatment of *ginnala* leaf extracts inhibited H_2O_2-induced oxidative stress. DCM and EA extracts also inhibited the cellular ROS production of NRCMs induced by 300μM H_2O_2. ROS intensity was determined by DCFDA assay. **p* < 0.05 versus H_2O_2. †*p* < 0.05 versus DMSO controls. *n*=4. Data represent the mean ± SEM.

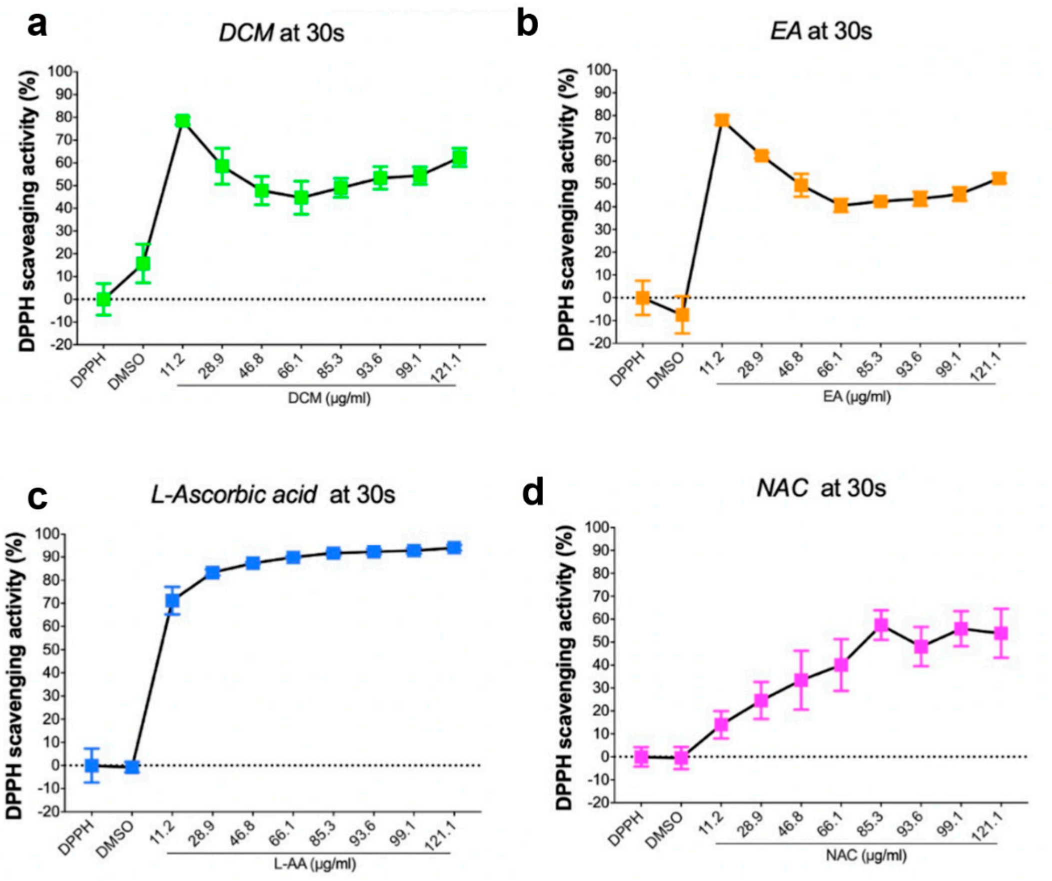

Supplementary Figure S4. Dose-dependent DPPH scavenging activities of *ginnala* leaf extracts and other common ROS scavengers. DPPH content was evaluated 30 s after mixing with different ROS scavenger including **(a)** DCM extracts, **(b)** EA extracts, **(c)** L-Ascorbic acid(L-AA) and **(d)** N-Acetylcysteine (NAC) with a series of concentration from 11.2 to 121.1μg/mL. *n*=3. Data represent the mean ± SEM.

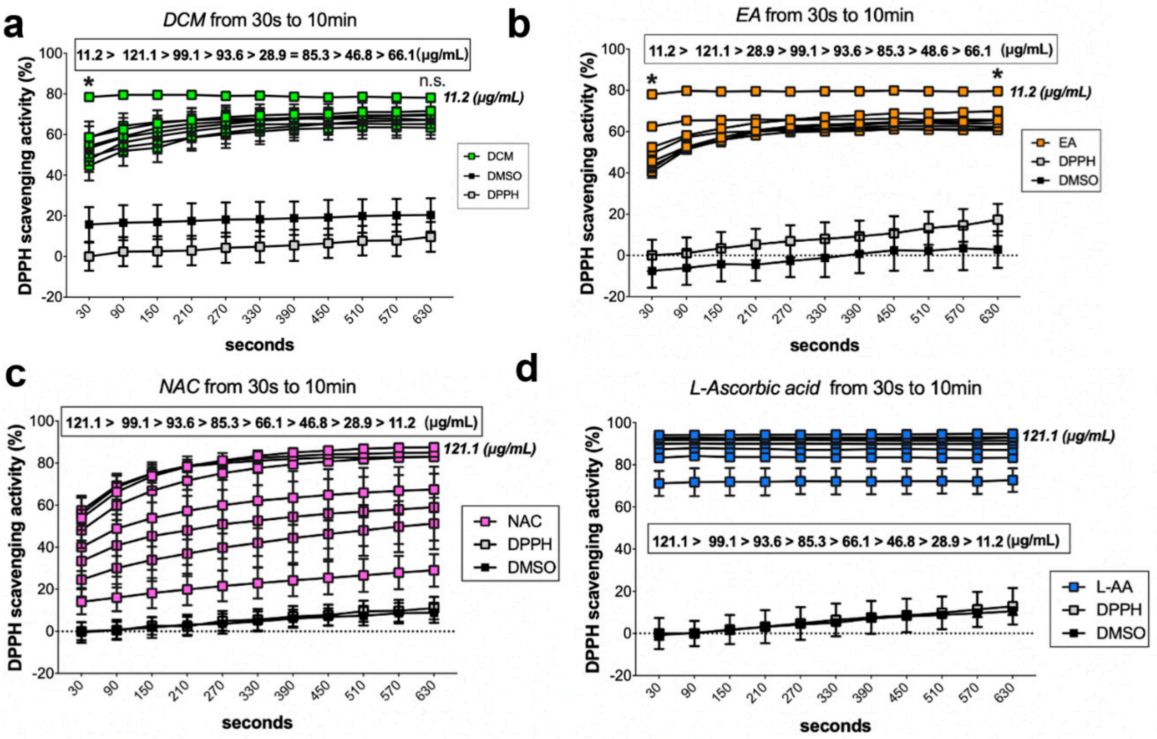

Supplementary Figure S5. Time-dependent DPPH scavenging activities of *ginnala* leaf extracts and other common ROS scavengers. DPPH content was evaluated at different time points from 30 s to 630 s after mixing with different ROS scavengers including **(a)** DCM extracts, **(b)** EA extracts, **(c)** N-Acetylcysteine (NAC) and **(d)** L-Ascorbic acid(L-AA) with a series of concentration. n=3. *$p < 0.05$ versus DCM or EA fractions at 66.1μg/mL. n=3. Data represent the mean ± SEM.

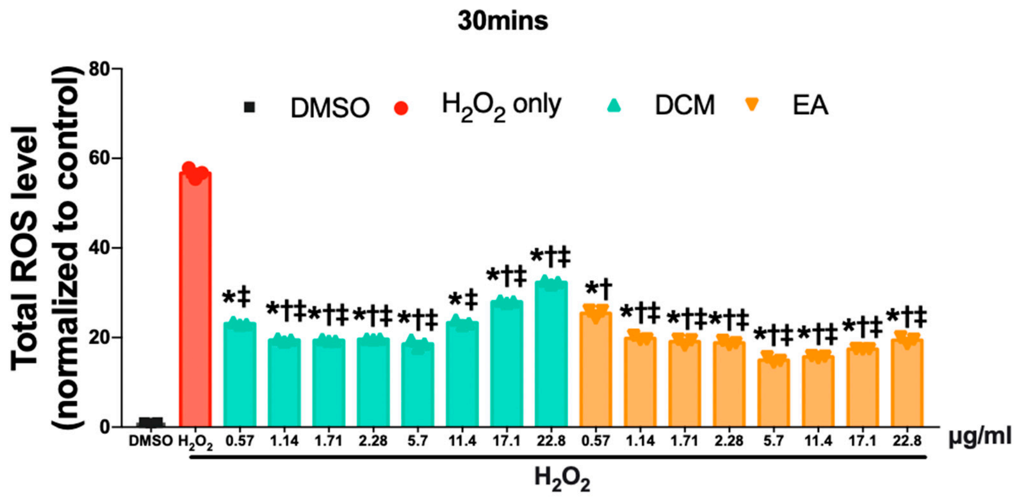

Supplementary Figure S6. ROS scavenging activities from *ginnala* leaf extracts were dose dependent. Total ROS level was determined by DCFDA assay 30min after mixing high concentration of H_2O_2(10mM) with different concentrations of DCM and EA extracts. *$p < 0.05$ versus H_2O_2; † $p < 0.05$ versus 0.57μg/mL DCM; ‡ $p < 0.05$ versus 0.57μg/mL EA. n=3. Data represent the mean ± SEM.

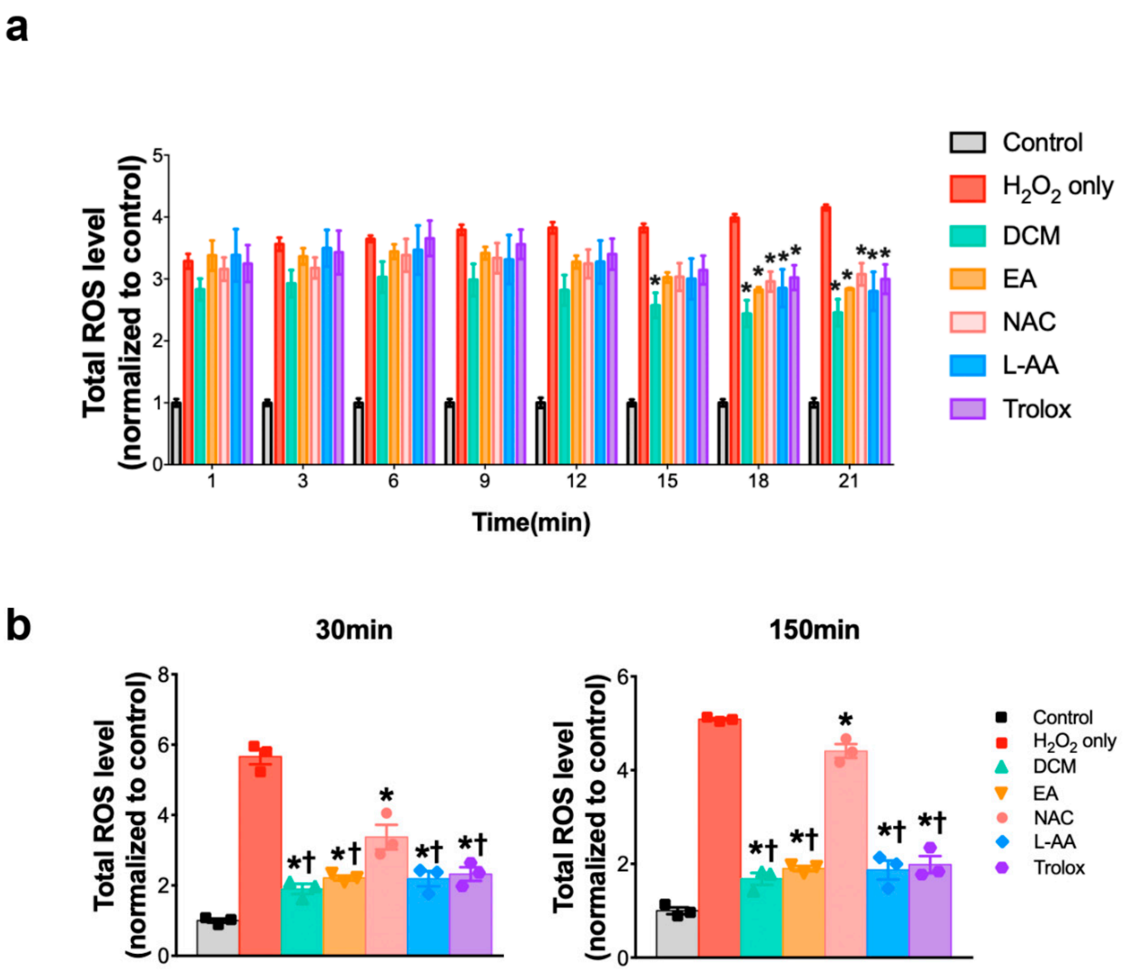

Supplementary Figure S7. *ginnala* leaf extracts exhibited faster intracellular ROS scavenging at same concentration of 46.8μg/mL. Intracellular ROS levels were evaluated from H_2O_2 treated NRCMs with post-incubation of ROS scavengers at the dose of 46.8μg/mL. **(a)** Tracing intracellular ROS levels 1~21min after treatment. **(b)** Tracing intracellular ROS levels at 30 and 150min after treatment. * $p < 0.05$ versus H_2O_2. † $p < 0.05$ versus NAC. n=3. Data represent the mean ± SEM.

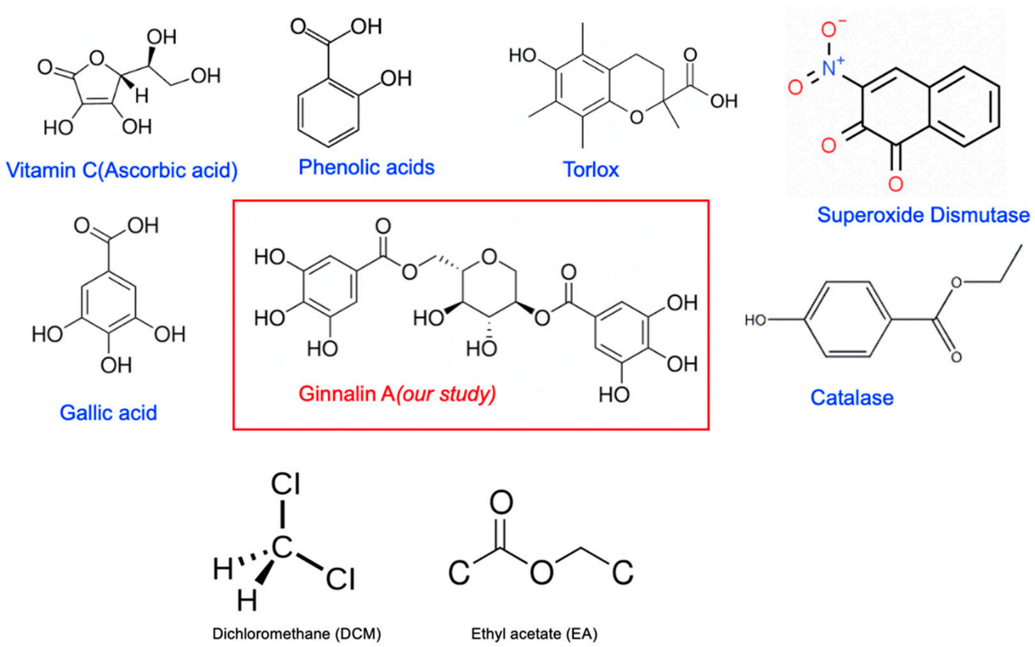

Supplementary Figure S8. Chemical structures of *Ginnalin A* and other common ROS scavengers.

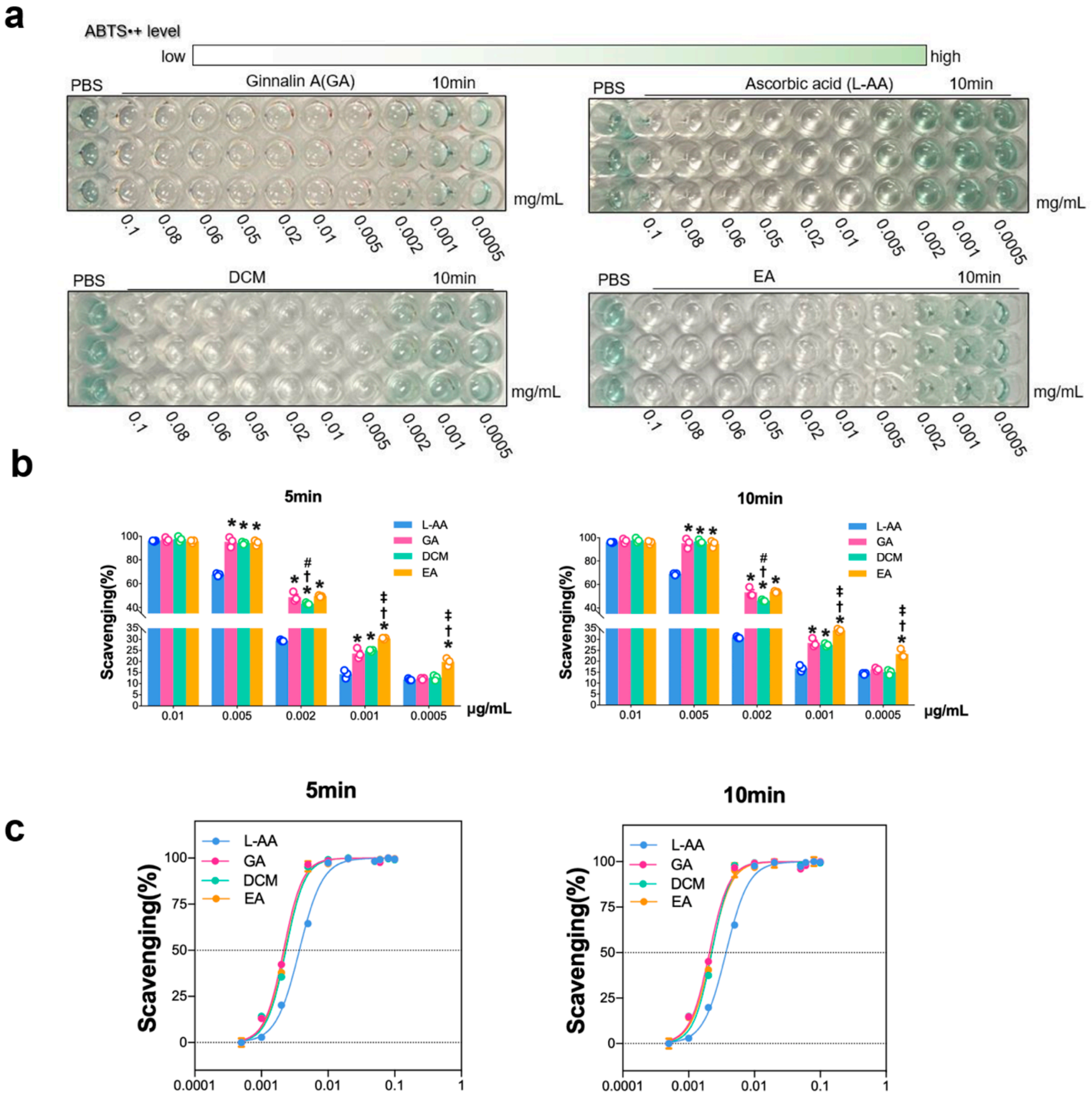

Supplementary Figure S9. ABTS $^{•+}$ scavenging effects from *ginnala* leaf extracts and L-AA at 5 and 10min. (a) Images depicted the ABTS$^{•+}$ scavenging activities of Ginnalin A(GA), L-Ascorbic acid(L-AA), DCM and EA at 10min. (**b**) ABTS •+ scavenging effects from *Ginnala* leaf extracts and L-AA with low doses. *$p < 0.05$ versus L-AA; †$p < 0.05$ versus GA; ‡ $p < 0.05$ versus DCM. n=3; # $p < 0.05$ versus EA. **(c)** Normalized ABTS$^{•+}$ scavenging curve after treating DCM, EA, GA and L-AA. n=3. Data represent the mean ± SEM.

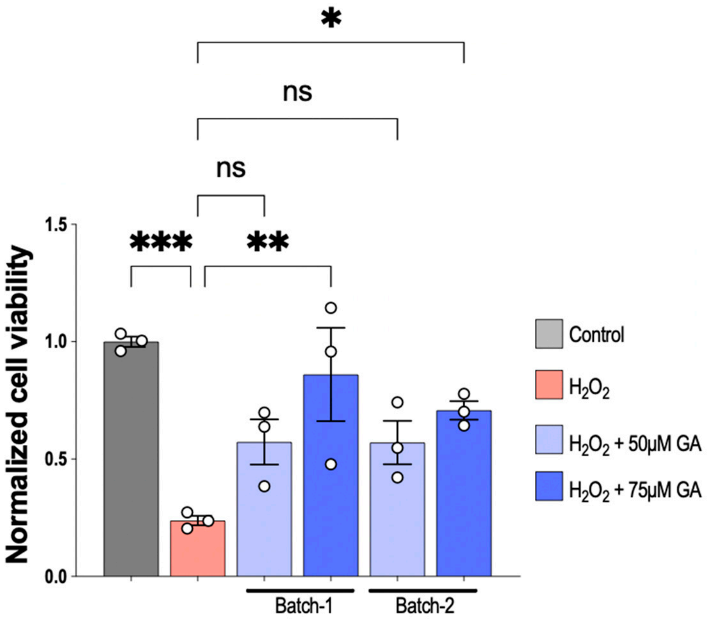

Supplementary Figure S10. ***Ginnalin A*****(GA) protected NRCMs against H_2O_2-induced cellular damage.** Cytoprotective effects of GA in 30min Co-treatment with 800μM H_2O_2 on neonatal rat cardiomyocytes (NRCMs) determined by CCK-8 assay. * $p < 0.05$, ** $p < 0.05$, *** $p < 0.05$ versus H_2O_2. n=3.

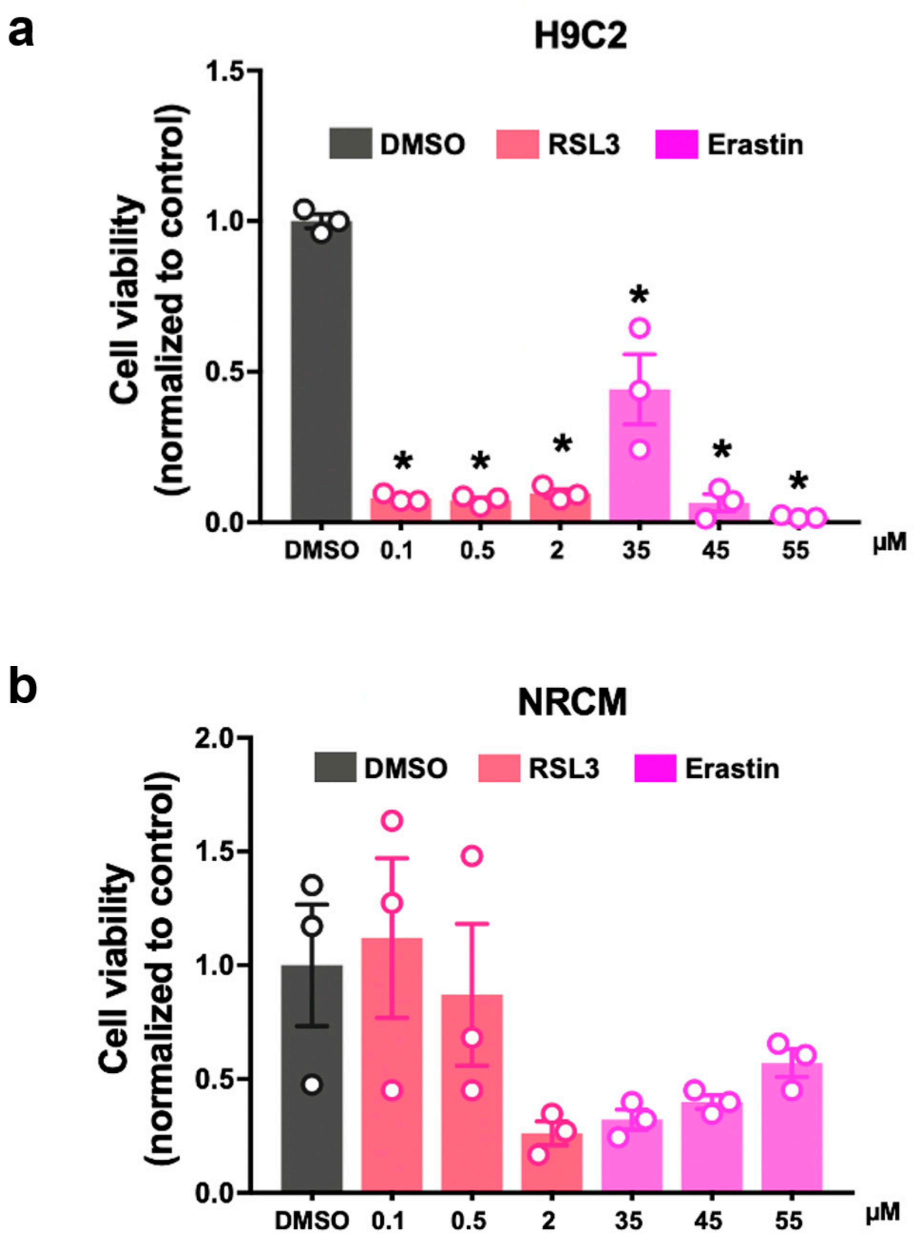

Supplementary Figure S11. Screening of Erastin and RSL3 from different concentration for inducing cell death of NRCMs. Screening cellular injury by treating different concentration Erastin and RSL3 on **(a)** H9C2 cells and **(b)** NRCMs. Cell viability was determined by CCK-8 assay. $*p < 0.05$ versus DMSO controls. n=3. Data represent the mean ± SEM.

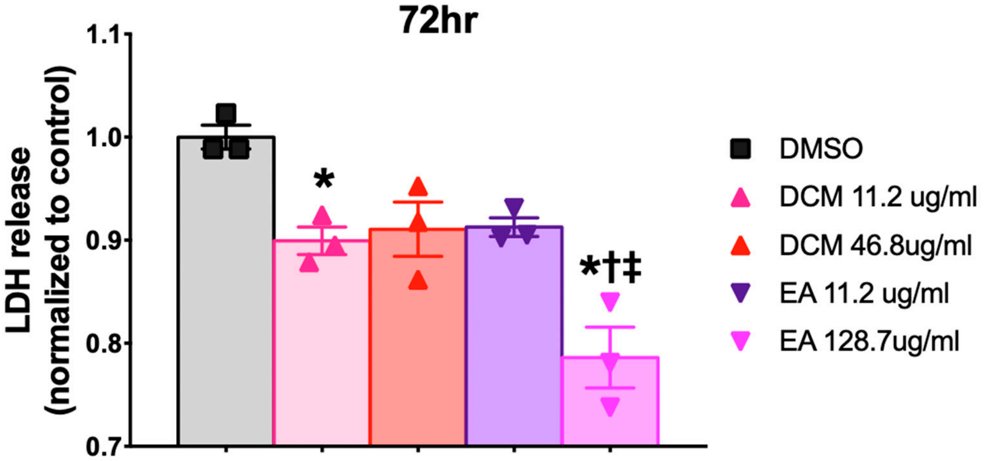

Supplementary Figure S12. Cellular toxicity evaluation of *ginnala* leaf extracts. Cellular damage was evaluated by LDH release after 72hrs administration DCM and EA fractions with different concentration. *$p < 0.05$ versus DMSO controls. †$p < 0.05$ versus 11.2μg/mL DCM; ‡ $p < 0.05$ versus 11.2μg/mL EA. n=3. Data represent the mean ± SEM.

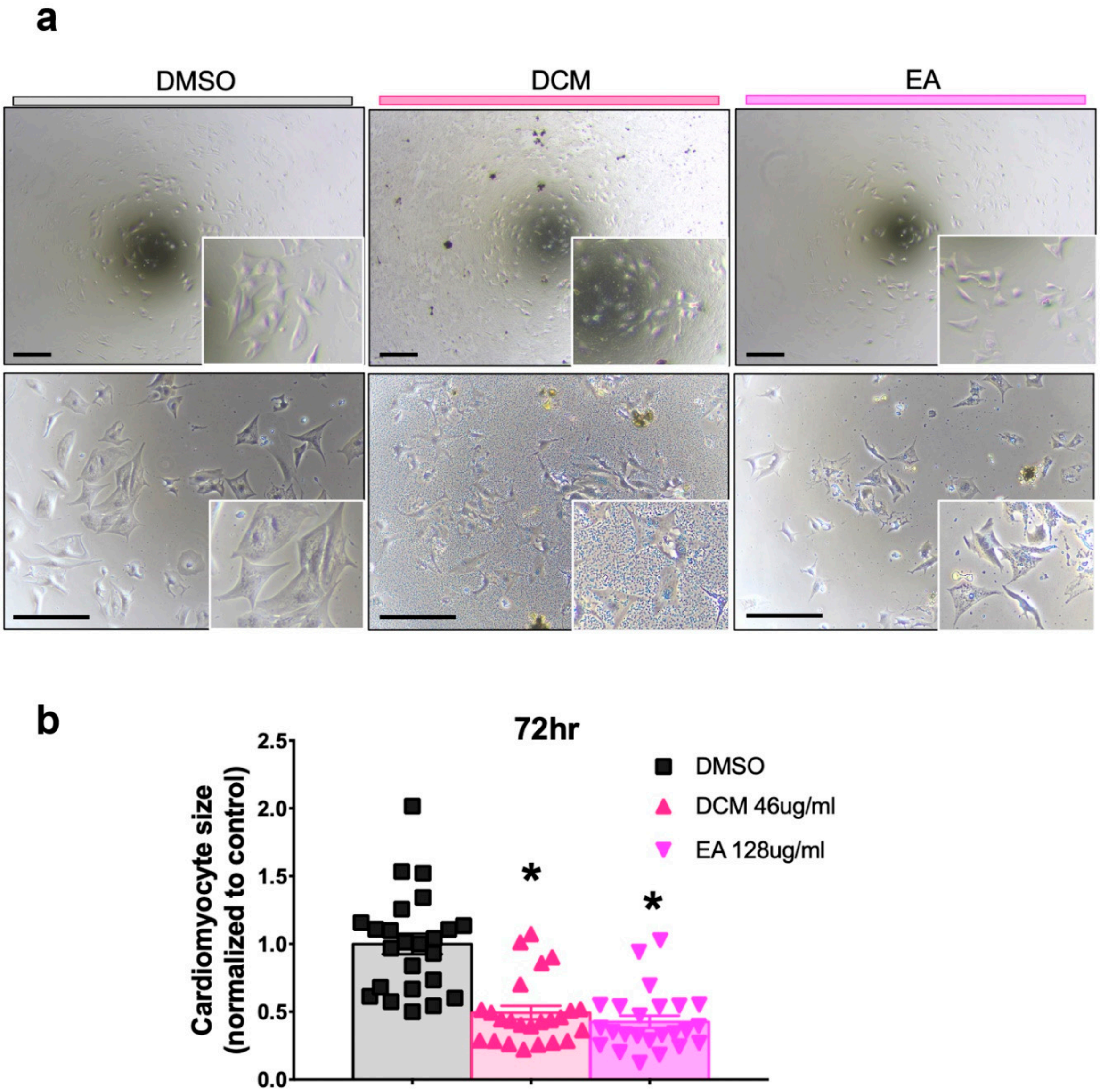

Supplementary Figure S13. *ginnala* leaf extracts prevented cardiomyocytes hypertrophy. (a) Images from 4x and 10x depicted the differential size of NRCMs and **(b)** following quantification analysis. *$p < 0.05$ versus DMSO. n=24. Scale bar=100μm. Data represent the mean ± SEM.

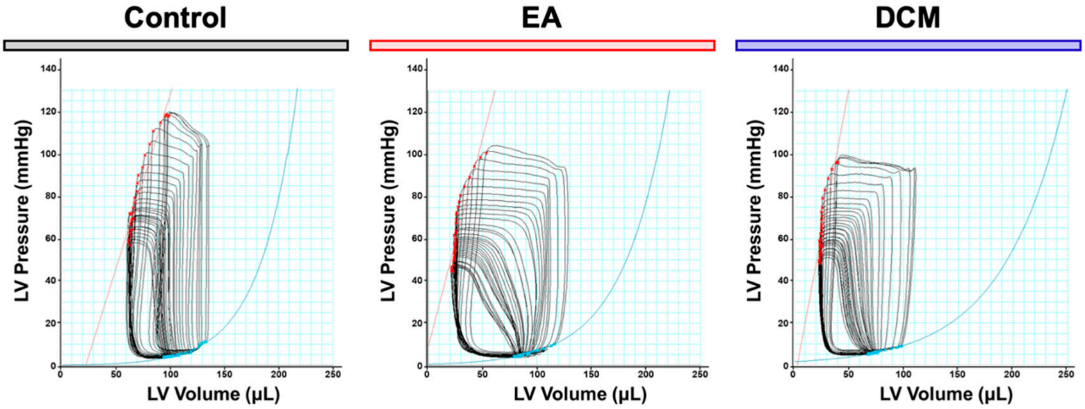

Supplementary Figure S14. Representative images of the slope of end-systolic pressure volume relationship (ESPVR). Images indicated the intrinsic cardiac contractibility as measured by transient inferior vena cava (IVC) occlusion, Slope of end-diastolic pressure volume relationship (EDPVR).
